# 3GPP NR V2X Mode 2: Overview, Models and System-Level Evaluation

**DOI:** 10.1109/access.2021.3090855

**Published:** 2021-06

**Authors:** ZORAZE ALI, SANDRA LAGÉN, LORENZA GIUPPONI, RICHARD ROUIL

**Affiliations:** 1 Centre Tecnològic de Telecomunicacions de Catalunya (CTTC/CERCA), 08860 Castelldefels, Spain; 2 National Institute of Standards and Technology (NIST), Gaithersburg, MD 20899, USA

**Keywords:** Vehicular communications, 3GPP, NR V2X, autonomous resource selection, network simulations

## Abstract

Following the successful use of sidelink in Long Term Evolution (LTE) for Proximity Services (ProSe) and Cellular Vehicular-to-everything (C-V2X), the 3rd Generation Partnership Project (3GPP) is working towards its evolution in New Radio (NR) systems in the context of the so-called NR V2X. This new technology is expected to complement LTE C-V2X for advanced services by offering low latency, high reliability, and high throughput V2X services for advanced driving use cases. To do this, NR V2X is equipped with new features, such as the support for groupcast and unicast communication, a novel feedback channel, and a new control channel design. In this paper, we provide a complete history of sidelink technology in 3GPP followed by a detailed overview of NR V2X technology, with special emphasis on Mode 2 for out of coverage operation and autonomous resource selection. Furthermore, this paper presents a system-level NR V2X standard-compliant simulator, as an extension of the popular and open-source NR network simulator 5G-LENA, based on ns-3. In particular, we focus on the design, implementation, and evaluation of the sensing-based resource selection in NR V2X Mode 2, in a highway scenario. Through several and extensive simulation campaigns, we test the impact of different NR V2X parameters, such as the numerology, the resource selection window size, the number of retransmissions, the maximum number of resources per reservation, and the probability of keeping the same resources during reselection, in a sensing-based resource selection. Finally, we provide a comparison campaign that shows the gains attained by the sensing-based resource selection, proposed during 3GPP Release 16, over the random selection strategy, considered in 3GPP Release 17 for power saving purposes.

## I. INTRODUCTION

The automotive industry is currently transitioning towards automated driving and advanced driver assisted systems, where vehicles are able to react by themselves to changes in the driving environment. In this context, Vehicular-to-everything (V2X) is seen as a key technology to provide complete environmental awareness around the vehicle by exchanging messages with other vehicles, roadside units, and pedestrians with low latency and high reliability. V2X communications are expected to provide potentiality in different areas, like faster alerts and notifications, law enforcement, better service on roadways, reduced world-wide traffic load, reduced emissions, time savings, and increased automotive safety, thus contributing to prevent crashes/injuries and save lives [1]. Additionally, V2X-capable vehicles can assist in better traffic management also for non-safety applications. Several advanced V2X use cases have been already proposed within the Third Generation Partnership Project (3GPP) Release 15 such as vehicle platooning, extended sensors, advanced and remote driving, or cooperative collision avoidance [2]. Also, industrial associations like the 5G Automotive Association (5GAA) in Europe have been built to promote the vision of connected mobility, including autonomous driving and intelligent transportation [3].

As of today, the two key radio access technologies that enable vehicular communications are 1) Dedicated Short Range Communications (DSRC), standardized by IEEE in 802.11p [4] and the more recent 802.11bd [5], and 2) Long Term Evolution (LTE) Cellular V2X (C-V2X), based on 3GPP LTE Release 14 and Release 15 [6]. DSRC is designed to primarily operate in the 5.9 GHz band, while C-V2X is thought to operate in both 5.9 GHz and in cellular licensed carriers at sub 6 GHz carrier frequencies. Differently from DSRC that focused on Vehicle-to-Vehicle (V2V) and Vehicle-to-Infrastructure (V2I) communications, C-V2X encompasses V2V, Vehicle-to-Pedestrian (V2P), V2I and Vehicle-to-Network (V2N) [7]. C-V2X is designed to support basic safety message sharing among proximity users, such as collision warning, emergency stop warning, and adaptive cruise control. A comparison study in [8] shows that LTE C-V2X gets a superior reliability performance over DSRC, due to the more efficient Physical (PHY) layer of LTE C-V2X.

V2X requirements can be met using LTE C-V2X, as long as the vehicular density is not too high [6]. However, as the quality of service requirements become more stringent, which is the case in many V2X applications, LTE C-V2X falls short, and Fifth-Generation (5G) New Radio (NR) is called as a complementary solution [5]. Towards that goal, 3GPP Release 16 has included a Study Item (SI) to support new applications with more stringent requirements, which has resulted in Technical Report (TR) 38.885 [9]. Based on the study outcome captured in this TR, 3GPP Release 16 has completed a Work Item (WI) in July 2020 to standardize V2X on top of 5G NR standardized in Release 15 [10]. As a main design principle, NR is not designed to be backward compatible with LTE. Similarly, NR V2X is not backward compatible with LTE C-V2X. The NR V2X SI indicates that the design objective of NR V2X is not to replace LTE C-V2X, but to supplement C-V2X in supporting those use cases that cannot be supported by LTE C-V2X [9]. To ensure that NR V2X can provide a unified support for all V2X applications in the future, NR V2X must be capable of supporting not only advanced V2X applications but also basic safety applications that are supported today by LTE C-V2X.

Such a wide applications and use cases’ support is possible in NR V2X because of the flexible framework inherited by the NR technology and the recent progresses envisioned in NR V2X standardization, which includes many enhancements over LTE C-V2X concepts. The NR radio access technology provides wide bandwidth support in various frequency ranges (including sub 6 GHz bands and millimeter-wave (mmWave) bands), flexible frame structure with reduced transmission time intervals (by means of multiple numerologies and sub-carrier spacing (SCS) support), support for massive Multiple-Input Multiple-Output (MIMO) systems and high modulation orders, and advanced channel coding [11]. All these new features and functionalities intrinsically contribute to increase the data rate, reduce the latency, and improve the spectral efficiency of V2X communication systems. In addition, new enhancements and key procedures have been defined for NR V2X, specifically designed to improve the reliability of V2X communications systems, such as new communication types (unicast and groupcast), a new feedback channel, the support of feedback-based retransmissions, and new resource allocation and scheduling mechanisms [9].

While LTE C-V2X has been widely studied analytically and through simulations by academia and industry [6], [8], [12]–[14], the studies on NR V2X have just started. Authors in [15] provide an overview of the standardization activities for vehicular communications at mmWave bands, including IEEE 802.11bd and 3GPP NR V2X specifications. Authors in [16] review the NR V2X design in 3GPP Release 16, with respect to the network architecture, security, and protocol enhancements. Authors in [17] provide a comprehensive overview of 3GPP NR sidelink transmissions, including physical layer structure, resource allocation mechanisms, and synchronization procedures. A more in-depth tutorial of 3GPP Release 16 NR V2X standard is presented in [18], including overview of the PHY layer, resource allocation, quality of service management, mobility management for V2N communications, and coexistence mechanisms between NR V2X and LTE C-V2X. In [19], the impact of the NR numerology on the V2X autonomous sidelink mode (similar to NR V2X Mode 2) is assessed. However, in [19], the evaluation is done over an LTE C-V2X simulator.

As it can be observed from the above review, most of the publicly available papers about NR V2X deal with a 3GPP standard overview, but few of these works discuss simulation studies and to the best of the authors’ knowledge none of them is based on Release 16 NR-compliant V2X simulation models, because the standardization has recently been completed. In addition, a key challenge to evaluate performance of NR V2X is that, despite the set of simulation results by industry and in literature, the simulators are not publicly available. Normally, simulators used in 3GPP are required to pass through a calibration procedure, but they are private, and not available to the research community. Consequently, the obtained results are nor reproducible, neither comparable, and system performance metrics are presented without much details revealed about the underlying models and assumptions. There are then private commercial simulators that are available after paying an annual license fee for using them. Often, if not in all cases, the license is very restrictive and does not allow modifications or inspection of the source code, which is a clear limitation for the research and the potential innovation. To the best of our knowledge, open source end-to-end simulators for 5G V2X communications compliant with NR V2X Release 16 specifications are not yet available to the research community.

There are five main open source and end-to-end simulators that have been developed to simulate sidelink communications. First, an LTE Device-to-Device (D2D) communication simulation model based on ns-3 was introduced and validated in [20]. Models are currently available through the ns-3 App store. Authors in [21] presented the first open-source simulator for LTE C-V2X Mode 4 communications, based on ns-3. An open-source 802.11p and LTE C-V2X simulation/emulation tool for ns-3, called ms-van3t, has been recently released in [22], which provides integration of ns-3 with the open-source Simulation of Urban MObility (SUMO) simulator for mobility management and mobility tracking. The work in [23] presents LTEV2Vsim, a simulator for LTE C-V2X Mode 3 and Mode 4 that is written in Matlab, freely available, and which focuses on Medium Access Control (MAC) and PHY layers procedures. Finally, authors in [24] introduced an ns-3 simulator for NR V2X at mmWave carrier frequencies. The model in [24] is compliant with 3GPP antenna and channel modeling for NR V2X, but not with NR V2X specifications at Radio Resource Control (RRC) and MAC layers. In particular, the model was developed before the finalization of NR V2X specifications. Therefore, at MAC layer it follows Mode 2 (c) for resource allocation, which was proposed as one of the options for study in 3GPP TR 38.885 [9]. Specifically, it uses Time Division Multiple Access (TDMA) to assign resources using the slots in a subframe, i.e., User Equipments (UEs) scheduled in a subframe have orthogonal resources to transmit on; hence, they do not collide. Moreover, the error model used at the PHY layer is more suited for LTE but not for NR. All these limitations have been addressed by our model as described below and throughout the paper.

In this paper, we consider the open source ns-3 5G-LENA simulator [25], as the basis for NR network simulation models, and we propose an extension to it to support NR V2X capabilities. In particular, we have focused on NR V2X Mode 2 communication, which is designed for out-of-coverage scenarios and direct V2V communications, in which the vehicles perform an autonomous resource selection without assistance of the base station. The 5G-LENA simulator and its extensions are available to download from [26]. In this paper, after reviewing the history of sidelink communications and the NR V2X standard with particular emphasis on NR V2X Mode 2, we present the proposed simulation tool and provide the implementation details of the developed NR V2X models, including the design choices and implementation changes that affect all the layers of the protocol stack. Based on these models, we present a comprehensive set of simulation campaigns, in which we study the impact on the end-to-end network performance of different key parameters of the NR V2X system when using sensing-based resource selection in NR V2X Mode 2, as defined by 3GPP. In particular, we study the impact of the numerology, the number of retransmissions, the length of the resource selection window, the maximum number of resources per reservation, the probability of keeping the same resource over multiple reservation periods, and the Modulation Coding Scheme (MCS). Finally, we compare different resource selection procedures for NR V2X Mode 2 considered in 3GPP, including sensing-based and random resource selections. From these detailed end-to-end campaigns, we derive interesting insights on the technology, which are summarized at the end of this work.

The rest of the paper is organized as follows. Section II reviews the history of sidelink communications in 3GPP and its various releases. Section III presents 3GPP NR V2X specifications and reviews in detail NR V2X Mode 2 transmissions. Section IV presents the simulation models and the implementation details. Section V discusses multiple simulation campaigns. Finally, Section VI concludes the paper.

## II. HISTORY OF SIDELINK TECHNOLOGY IN 3GPP

The concept of sidelink was first introduced in Release 12, together with D2D communications extensions to the traditionally centralized paradigm of cellular communications promoted by 3GPP. LTEC-V2X first and NR V2X later are all significantly based on previous D2D efforts [27], [28]. In this section we review the history of D2D and V2X technologies inside 3GPP, giving special emphasis to Releases 12, 14, and 16, which are those starting the definition of the new D2D, C-V2X, and NR V2X technologies, respectively.

Tables 1 and 2 summarize the evolution of sidelink communications in 3GPP, since its introduction with D2D (Release 12/13), through LTE C-V2X (Release 14/15) and up to date in NR V2X (Release 16/17), including various SIs and WIs related to sidelink communication studies and standardization. For each SI/WI, we specify the 3GPP release, the working/leading group in charge of such SI/WI, the objective of the SI/WI, the resulting TR for the case of SIs and the impacted Technical Specification (TS) for the case of WIs. The history of sidelink is split into two tables. Table 1 covers from Release 12 until Release 15 and Table 2 covers the SIs/WIs from Release 16 until Release 17.

### A. D2D (RELEASE 12)

D2D has been defined as a support for Proximity Services (ProSe). D2D enables the quick exchange of data over short distances via a direct link between nodes and introduces a new interface, the PC5, between nodes. This offers an efficient way to bypass the LTE base station (or Evolved Node B (eNB)) and offload the eNB traffic. Besides content sharing, a D2D UE can act as a relay for another device with a poor connection to the eNB and, therefore, D2D can be used to extend cellular network coverage. Two modes have been defined for centralized and distributed scheduling of UE transmissions, namely Mode 1 and Mode 2. Centralized scheduling occurs at the eNB (in-coverage mode), whereas distributed scheduling is carried out by the D2D UEs themselves, with no need to be in the coverage area of an eNB (out-of-coverage mode). In Mode 1, the UEs are scheduled by the eNB over dedicated radio resources for data transmission. In Mode 2, a UE can autonomously select a radio resource from a resource pool, which is either configured by the network or pre-configured in the user device for its direct D2D communication over PC5 interface.

Both modes share the same resource allocation structure, in which the transmission of data is scheduled within the so-called sidelink control period. Within this period, a set of subframes are allocated for the Physical Sidelink Control Channel (PSCCH) transmission and a different set of subframes are allocated for the Physical Sidelink Shared Channel (PSSCH). The corresponding PSCCH for a given PSSCH is always sent before the PSSCH data. The PSCCH contains the Sidelink Control Information (SCI), also called scheduling assignment, which is used by the receiver to identify the occupation of the PSSCH radio resources. In both modes, the SCI is configured in format 0, and it is transmitted twice using two different subframes in which it occupies the same Resource Block (RB). The second transmission is needed to improve the reliability of the SCI message delivery at the receiver due to the lack of a feedback channel in sidelink communication. The receiver blindly detects the SCI by monitoring all possible PSCCH resources. The transport block is transmitted four times in four consecutive subframes within the resource pool. This allows the receiver UE to implement open loop Hybrid Automatic Repeat Request (HARQ) by combining the four redundancy versions of the transport block.

The operational principle of Modes 1 and 2 is battery life improvement of mobile devices. Vehicular communications have, however, other constraints that cannot be accommodated with D2D ProSe. Specifically, the high latencies of D2D are not suitable for vehicular communications, where packet delays or packet losses can have severe and life-threatening consequences. In terms of requirements, the maximum allowed latency varies between 20 ms and 100 ms, depending on the application, with reliability from 80 % to 95 % [7].

### B. LTE C-V2X (RELEASE 14)

3GPP Release 14 extended the D2D ProSe functionality by adding two new modes, Modes 3 and 4, for LTE C-V2X connectivity. Basic safety messages and event-triggered messages are transmitted for collision avoidance. V2V mainly enables cooperative automated driving. V2P establishes the communications protocol between vehicles and pedestrians for pedestrian safety. V2I implies the communications with roadside units and allows to make information about local road and traffic conditions readily available to vehicles. V2N enables commercial services by providing access to data stored in the Cloud.

Modes 3 and 4 have been designed to satisfy the latency requirements and accommodate high Doppler spreads and high density of vehicles for LTE C-V2X communications. Similarly to Mode 1, Mode 3 uses the centralized eNB scheduler. The vehicular UE and eNB use the Uu interface to communicate. This transmission mode is only available when the vehicles are under cellular coverage. UE context information in terms of traffic patterns, for example, can be reported to the eNBs in order to assist in the resource allocation procedure. Mode 4 employs distributed UE scheduling, as Mode 2. In contrast to Mode 3, Mode 4 can operate without cellular coverage. However, these modes share a completely different structure than Modes 1 and 2 described above, when it comes to the allocation of the PSCCH and PSSCH. First, PSCCH and PSSCH channels are not separated in the temporal domain, but in the frequency domain. The resource grid is divided into sub-bands or sub-channels in which the first RBs of these sub-channels form the PSCCH pool and, the other RBs, the PSSCH pool. A new SCI format, format 1, is employed. In Modes 3 and 4, a transport block can be sent either once or twice. In case of two transmitting attempts, the information is sent over another subframe, with the same structure: two SCIs and their corresponding PSSCH transport block. In this case, the receiver also implements HARQ combining. Vehicles select their sub-channels in Mode 4 using the sensing-based Semi-Persistent Scheduling (SPS) scheme specified in Release 14. Thanks to the semi-persistent reservation of resources and the inclusion of the reselection counter and packet transmission interval in the SCI, other vehicles can estimate which subchannels/subframes are free when making their own reservation, which reduces packet collisions.

### C. NR V2X (RELEASE 16)

To support a wide range of V2X applications with different quality of service requirements and support scenarios with high vehicular density, 3GPP has continued the standardization efforts on V2X communications through NR V2X in Release 16 and 17.

The requirements agreed for 5G V2X services and to be met by 3GPP standards are described in [2], where design requirements for 25 different 5G V2X use cases are presented. Thanks to the flexibility provided by 5G NR and the recent progresses envisioned in NR V2X, the support for a wide range of applications is feasible with NR V2X technology. The initial NR V2X design was developed in NR Release 16 SI [9], and was then included in the NR Release 16 specification based on the NR V2X WI [10]. Like IEEE 802.11bd and 5G NR, NR V2X also considers the use of mmWave bands for V2X applications, particularly for applications that require a short range and high to very high throughputs. However, considering the limited timeline of 3GPP Release 16, NR V2X mmWave operations were deprioritized in the 3GPP WI [10]. In this line, TR 38.885 conducted a limited study on beam management and concluded that it is beneficial for sidelink, but also that in sub 6 GHz bands it is feasible to support V2X use cases without sidelink beam management. Table 3 presents the sub 6 GHz operating bands for NR V2X, which is obtained from tables in [29] and [30].

The services specified for NR V2X range between 25 Mbit/s and 1 Gbit/s for data rate, 90 % to 99.99 % for reliability, and 5 ms to 100 ms for latency, depending on the use case [7]. Those latency requirements can not be met by Release 14 LTE C-V2X, but they can be improved considering higher numerologies in NR. Also, the reliability requirement of 99.99 % requires that NR V2X standardizes new enhancements at both resource allocation and scheduling. Extensive details of NR V2X will be given in the next Section III.

## III. NR V2X TECHNOLOGY REVIEW

This section presents the main highlights of NR V2X technology in 3GPP, with special emphasis to NR V2X Mode 2 transmissions.

### A. NR V2X

#### 1) COMMUNICATION TYPES

Differently from LTE C-V2X that focused on periodic basic safety messages, NR V2X has been designed to support various use cases, including the transmission of periodic traffic as well as reliable delivery of aperiodic messages. To support a wide range of different new applications, NR V2X goes beyond the only broadcast communications proposed by LTE C-V2X, and provides support for three types of transmissions: broadcast, groupcast, and unicast [2], [9]. In NR V2X unicast transmissions, the transmitting UE has a single receiver UE associated with it. The groupcast mode is used when the transmitting UE wishes to communicate with a specific sub-set of UEs in its vicinity. Finally, broadcast transmissions enable a UE to communicate with all UEs within its transmission range. In NR V2X, a single UE can establish communications of multiple types simultaneously. For example, a platoon leader UE can communicate with its platoon member UEs using the groupcast mode, while using the broadcast mode to transmit other periodic messages to UEs that are not part of the platoon.

#### 2) SIDELINK PHYSICAL CHANNELS AND REFERENCE SIGNALS

Sidelink communications in NR V2X use the following physical channels [31]: 1) the Physical Sidelink Broadcast Channel (PSBCH) for sending broadcast information (like synchronization of the sidelink), 2) the PSCCH for sending control information (1st-stage-SCI), 3) the PSSCH for sending control (2nd-stage-SCI), data and Channel State Information (CSI) in case of unicast, 4) and the Physical Sidelink Feedback Channel (PSFCH) for sending HARQ feedback in case of unicast and groupcast modes. The PSFCH is a new channel, which was not previously considered in LTE C-V2X. For these channels, numerologies 0 (SCS=15 kHz), 1 (SCS=30 kHz), and 2 (SCS=60 kHz) are supported at sub 6 GHz bands, and numerologies 2 (SCS=60 kHz) and 3 (SCS=120 kHz) can be used at mmWave bands [32]. For PSSCH, the supported modulation schemes include QPSK, 16-QAM, 64-QAM, and 256-QAM. Instead, for PSCCH, only QPSK transmission is supported.

Regarding the reference signals, NR V2X uses [17] 1) the Sidelink Primary/Secondary Synchronization Signal (S-PSS/S-SSS) for synchronization. S-PSS/S-SSS are transmitted together with the PSBCH in the so-called synchronization signal/PSBCH block (SSB). The SSB uses the same numerology as the PSCCH/PSSCH on that carrier. 2) Demodulation Reference Signals (DMRS) to estimate the channel and perform data decoding. 3) Phase Tracking Reference Signal (PT-RS) to compensate for phase noise. 4) Channel State Information Reference Signal (CSI-RS) to estimate the channel and report channel quality information, similarly to NR.

#### 3) SIDELINK RESOURCE POOL

An important aspect of sidelink communications is the definition of sidelink resource pools. In NR V2X, a UE can be configured by higher layers with one or more sidelink resource pools. A sidelink resource pool can be used for transmission and reception of PSCCH/PSSCH, and can be associated with either sidelink resource allocation Mode 1 or Mode 2 [9]. In the frequency domain, a sidelink resource pool consists of a number of contiguous subchannels [33]. The size of each subchannel is fixed and it is composed of *N* contiguous RBs. Both the number of subchannels and the subchannel size are higher layer pre-configured, by RRC. NR V2X supports *N* = 10, 15, 20, 25, 50, 75, and 100 RBs for possible sub-channel sizes [34]. In the time domain, the resources (i.e., slots) available for sidelink are determined by repeating sidelink bitmaps. The bitmap is pre-configured and characterized by a certain size. The resource pool parameter from RRC, sl-TimeResource, defines the bitmap size and takes values 10, 11, 12, ..., 160 [35]. In particular, in case of Time Division Duplex (TDD), the resources available for sidelink are given by the combination of the TDD pattern and the sidelink bitmap. We also note that, unlike LTE sidelink specification related to the TDD pattern and the size of sidelink bitmap [36], NR sidelink specification is flexible and any valid NR TDD pattern can be used with any structure of a sidelink bitmap, which has a size specified by the standard [37]. Since NR V2X may be developed both in a carrier dedicated to Intelligent Transport System (ITS) or to cellular services, the standards support both the cases where all the symbols in a slot are available for sidelink, or only a consecutive subset of them [9]. In ITS spectrum, all the symbols are always allocated to sidelink. Within the slots available for sidelink, the specific Orthogonal Frequency Division Multiplexing (OFDM) symbols used for sidelink transmission/reception are fixed and pre-configured. Two RRC parameters pre-configure the symbol index of the first symbol and the set of consecutive symbols in a slot available for sidelink [33].

**FIGURE 1.**
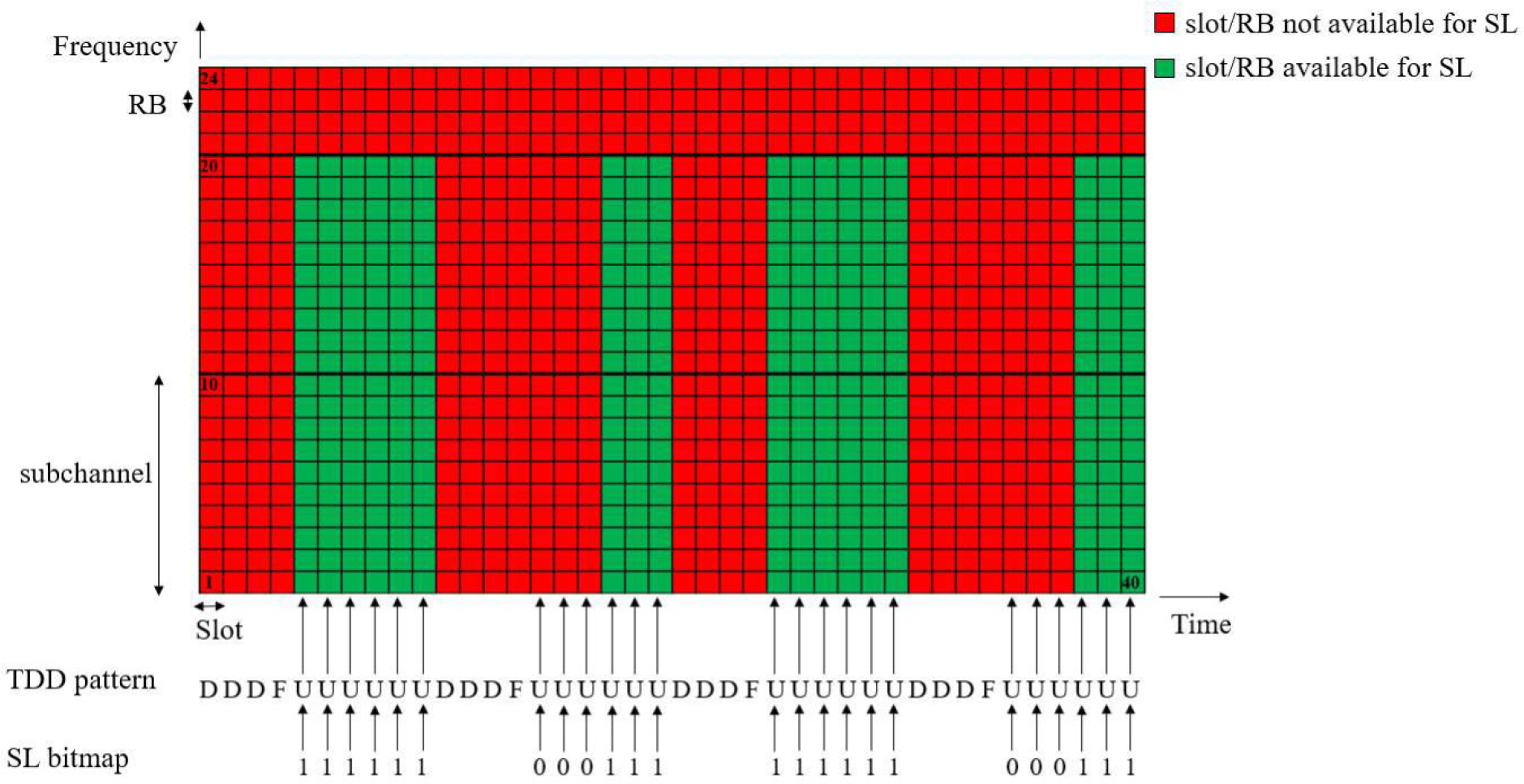
Time/frequency frame structure and definition of sidelink resource pool for NR V2X TDD. Example with 2 subchannels of 10 RBs each, using TDD pattern of [D D D F U U U U U U] and sidelink bitmap of [1 1 1 1 1 1 0 0 0 1 1 1].

In Fig. 1 we illustrate the time/frequency frame structure of NR V2X and the definition of sidelink resource pools for TDD systems. The example is shown for the case of 10 MHz bandwidth using numerology 1 (i.e., SCS 30 kHz), and 2 subchannels, each composed of 10 RBs where RB 1 is the starting RB of the first sidelink subchannel. In time, we consider a TDD pattern of [D D D F U U U U U U] (i.e., one downlink slot, followed by a flexible slot,^1^ and three uplink slots), and a sidelink bitmap of [1 1 1 1 1 1 0 0 0 1 1 1]. As it can be observed, the TDD pattern is repeated in time, and each index of the sidelink bitmap applies to the uplink slots (U) in the TDD pattern, repeatedly, thus indicating the slots available for sidelink. In the frequency domain, a sidelink resource pool consists of a number of contiguous subchannels [33], therefore, as per [35], the last 4 RBs are not available for sidelink. As a result, in the figure we illustrate in green which slots/RBs are available for sidelink communications in the mentioned configuration example. This structure is typically used by an out-of-coverage NR V2X UE using Mode 2 operating in any of the V2X bands listed in Table 3. On the other hand, an in-coverage NR V2X UE operating in either Mode 1 or Mode 2, will tailor its time/frequency structure as per the next-Generation Node B (gNB) provided TDD pattern, sidelink bitmap, and subchannels.

#### 4) RETRANSMISSIONS AND NEW SIDELINK FEEDBACK CHANNEL

Differently from LTE C-V2X, which uses fixed MCS and only provides support for blind retransmissions, i.e., the source UE, automatically retransmits without knowing if the initial transmission has been correctly received, NR V2X provides different enhancements to improve reliability of communications, by introducing a feedback channel, the PSFCH. In particular, for unicast and groupcast communications considered by NR V2X, but not by LTE C-V2X, reliability can be improved if the source UE can retransmit the packet once the reception fails at the receiving UE and if the MCS can be adjusted to the actual channel conditions. NR-V2X introduces both blind and feedback-based retransmissions, for unicast and groupcast communications, while for broadcast communications only blind retransmissions are supported. With blind retransmissions, HARQ is implemented only at the receiver for retransmission combining. The transmitting UE chooses the resources within a resource reservation interval to retransmit. In particular the UE retransmits based on the configured value, which can be up to 31. Blind retransmissions are resource inefficient if the initial transmission is successful. On the other hand, feedback-based retransmissions are more resource efficient because the transmitting UE only retransmits if the original transmission is NACKed. In this case, HARQ is implemented both at the transmitter for efficient retransmissions and at the receiver for retransmission combining. In both cases, NR V2X Mode 2 supports a maximum number of PSSCH transmissions of the same MAC Packet Data Unit (PDU), which is pre-configured and whose maximum value is equal to 32. Even if feedback-based retransmissions are more resource efficient, blind retransmissions allow to minimize the latency of the feedback-based retransmissions, as the transmitting UE does not need to wait for a HARQ feedback before sending a retransmission [17]. To enable feedback-based retransmissions, NR V2X introduces the PSFCH.

#### 5) MULTIPLEXING OF PSCCH, PSSCH, AND PSFCH

In LTE C-V2X, PSCCH and PSSCH channels are multiplexed in the frequency domain. The drawback of this approach is that a receiver must buffer the message for the entire sub-frame and can decode the message only at the end of the sub-frame. This may be inefficient in NR V2X due to tight latency constraints of certain messages. To address this problem, different multiplexing options are considered in NR V2X for PSCCH and PSSCH [9]. Among the different options, two out of four consider time multiplexing in NR V2X, i.e., the PSCCH will be transmitted first, followed by the transmission of PSSCH. In time domain, within the symbols available for sidelink in a slot (see description in Section III-A3), the PSCCH can span over two or three symbols at the beginning of the pre-configured symbols and the PSSCH spans over the remaining number of pre-configured symbols. Finally, both PSCCH and PSSCH are multiplexed in time with the PSFCH. Specifically, every one, two, or four slots available for sidelink, the last two symbols among the pre-configured ones for sidelink, excluding the guard period symbol, are reserved for the PSFCH. In the frequency domain, the PSSCH can occupy up to the maximum number of available subchannels for sidelink, depending on the amount of data to transmit. However, the PSCCH spans over a pre-configured number of consecutive RBs (i.e., *K* RBs) in the first subchannel in which PSSCH is transmitted, where *K* ≤ *N* RBs and *N* is the subchannel size, described in Section III-A3. NR V2X supports *K* = 10, 12, 15, 20, and 25. Finally, the candidate resources, i.e., RBs for PSFCH are determined as per the PSSCH transmission(s) for which the feedback is generated. For more details, the reader is referred to very comprehensive tutorial of the NR V2X standard in [17] and [38].

**FIGURE 2.**
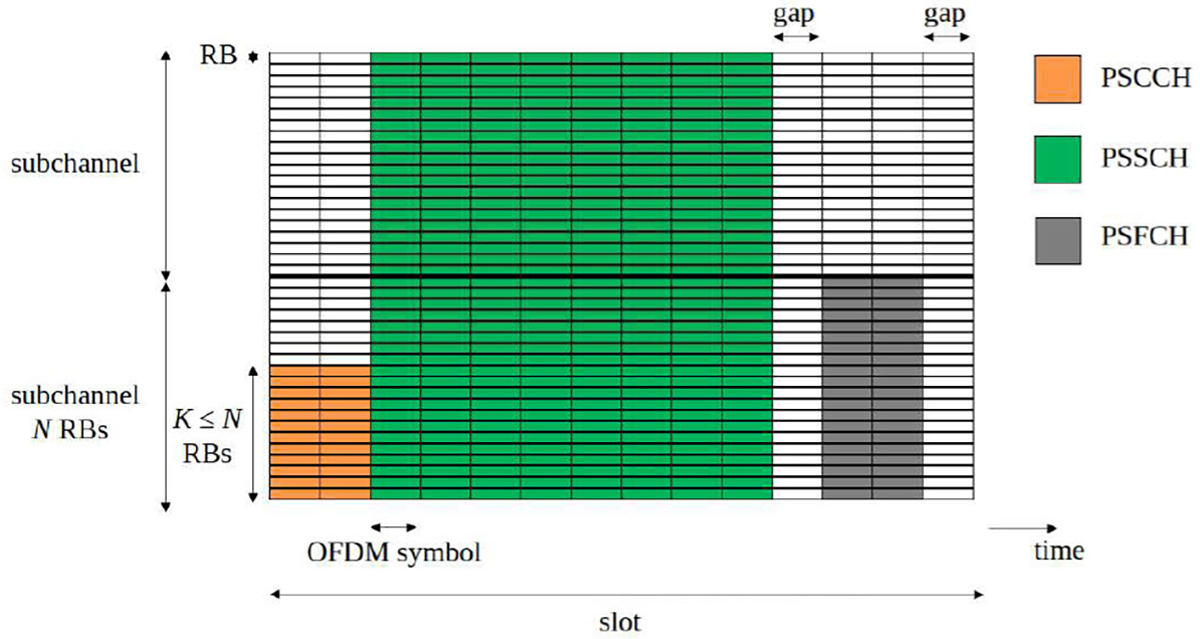
Slot structure of a slot available for sidelink, with time multiplexing of PSCCH, PSSCH, and PSFCH in NR V2X.

The time multiplexing of the PSCCH, PSSCH, and PSFCH in NR V2X is shown in Fig. 2, assuming a typical NR slot structure composed of 14 OFDM symbols. As previously mentioned, the number of OFDM symbols used for sidelink is pre-configured. In the example in Fig. 2, 14 symbols are available for sidelink, the length of PSCCH is pre-configured to 2 symbols, PSSCH starts at the 3rd symbol with a duration of 8 symbols, *N* = 20 RBs is the subchannel size,and *K* = 12 RBs are used for the PSCCH.

#### 6) RESOURCE ALLOCATION

NR V2X defines two resource allocation modes for sidelink communications, one centralized and one distributed [9]:

- Mode 1: The NR base station (gNB) schedules sidelink resources to be used by the UE for sidelink transmissions.
- Mode 2: The UE autonomously determines sidelink transmission resources within sidelink resources configured by the gNB or pre-configured by the network.

These two NR V2X modes are similar to LTE C-V2X Mode 3 and Mode 4, respectively. NR V2X Mode 1 is a centralized scheduling approach, in which the resource allocation is managed by the gNB and applies to scenarios in which the various UEs are inside the coverage of the gNB (i.e., in-coverage scenarios). On the other hand, NR V2X Mode 2 is a distributed scheduling approach in which the resource allocation is carried out by the UEs themselves, with no need to be in the coverage area of the gNB (i.e., it supports out-of-coverage communication). In this paper, we focus on NR V2X Mode 2 with periodic traffic.

Resource reservation for NR V2X Mode 2 under periodic traffic mostly reuses the LTE C-V2X sidelink Mode 4 long-term sensing-based algorithm. It exploits the periodicity and fixed-size assumption of basic safety messages. In addition to the long-term sensing-based resource selection, NR V2X Mode 2 also supports a random resource selection [39]. The difference between sensing-based and random resource selections is that, before selecting the resources from the total available ones, the sensing-based procedure filters those slots which are in use by other UEs, using sensing information. On the other hand, the random selection procedure does not use the sensing information, and directly selects the resources from the total available ones. The random resource selection approach is considered to reduce the complexity of the UE and the power consumption, since the sensing procedure adds complexity and has an energy cost at the transmitting UE that has to continuously sense the channel for resource selection. In particular, this random procedure is currently being addressed in an approved 3GPP NR Release 17 WI [40], as a power saving mechanism, which is especially relevant for the use cases of public safety and pedestrian UEs in V2X scenarios, where UEs have battery limited capacity and must operate efficiently. On the other hand, in case of aperiodic traffic, LTE C-V2X Mode 4 resource selection mechanism has been re-engineered for NR V2X Mode 2, since the arrival of future packets cannot be inferred by sensing previous transmissions from surrounding UEs. For these cases, the use of short-term sensing and dynamic reservation is envisioned in 3GPP [9].

#### 7) SIDELINK CONTROL INFORMATION

Another key improvement of NR V2X is the split of the SCI. Two SCI formats have been defined [41]: SCI Format 0–1 and SCI Format 0–2, which are sent through different channels, the PSCCH and the PSSCH, respectively. SCI carried on PSCCH is a 1st-stage SCI (SCI Format 0–1), which transports sidelink scheduling information of PSSCH and 2nd-stage-SCI on PSSCH. This sidelink scheduling information includes the priority, time/frequency resource assignment, 2nd-stage-SCI format, MCS, and resource reservation period. The SCI carried on PSSCH is a 2nd-stage-SCI (SCI Format 0–2), which transports information used for the decoding of PSSCH. This includes the HARQ process ID, new data indicator (NDI), redundancy version, source ID, and destination ID.

1st-stage-SCI indicates the reservation of $N_{\max\_\text{reserve}}$ (pre-configured) number of sidelink resources within the resource selection window [42]. $N_{\max\_\text{reserve}}$ can be 2 or 3 [35]. The resource reservation is indicated in the time resource assignment field of the 1st-stage-SCI. This means that not all the slots in a resource reservation period of a UE carry 1st-stage SCI in the PSCCH; some slots have empty PSCCH and only carry information in the PSSCH, as indicated by a 1st-stage-SCI in a previous slot.

**FIGURE 3.**
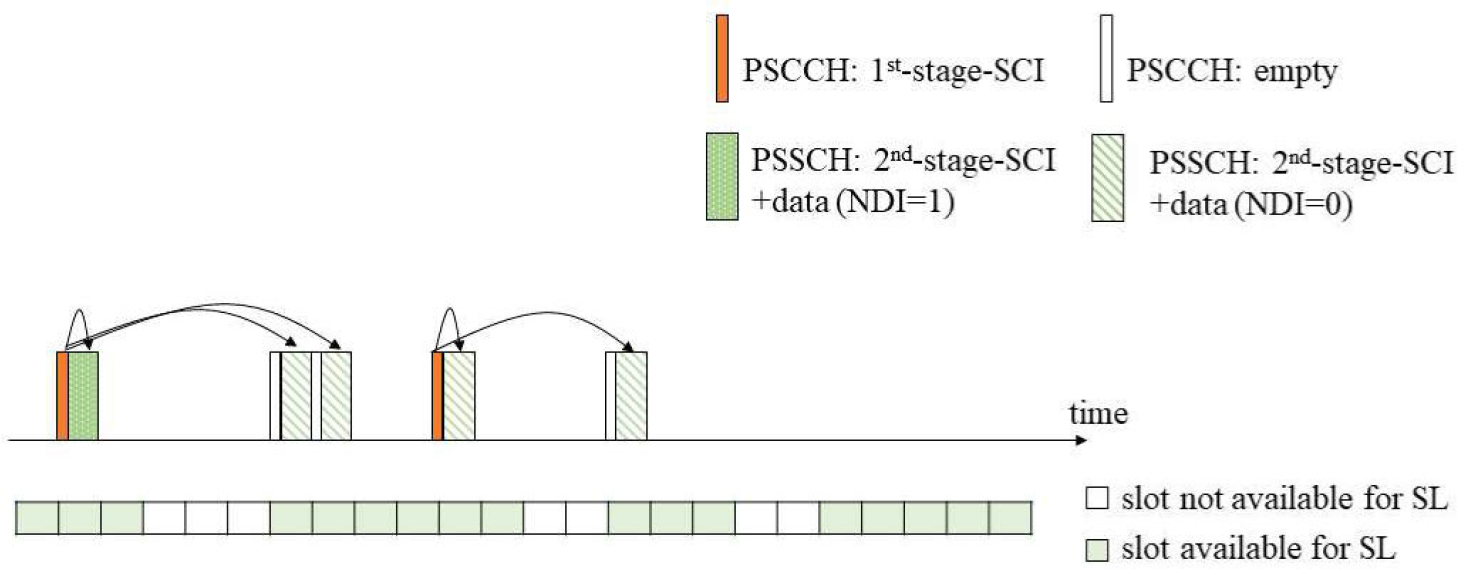
Illustration of the SCI split and resource reservation concept in NR V2X.

An illustration of the SCI split and the resource reservation mechanism is shown in Fig. 3, for the case of $N_{\max\_\text{reserve}}\;=\;3$ (i.e., each 1st-stage SCI can indicate up to three sidelink PSSCH resources) and $N_{\text{selected}}\;=\;5$. $N_{\text{selected}}$ indicates the number of resources that are selected within a selection window, as per [42]. As an example, in NR V2X Mode 2, upon a resource selection trigger, the UE MAC can select various resources for an initial transmission (NDI = 1) and various retransmissions (NDI = 0). According to NR V2X specification, $N_{\text{sci}}=\min\left(N_{\max\_\text{reserve}},\;N_{\text{selected}}\right)$ is the number of resources indicated by a 1st-stage-SCI [33]. In Fig. 3, *N*_sci_ = 3 for the first 1st-stage-SCI and *N*_sci_ = 2 for the second 1st-stage SCI (since here, only two remaining slots are left to be indicated after the resources indicated by the first 1st-stage-SCI).

### B. NR V2X MODE 2

NR V2X Mode 2 considers sensing-based SPS for periodic traffic. This is defined as a distributed scheduling protocol to autonomously select radio resources, in a similar way to what is already considered for LTE C-V2X Mode 4. The sensing procedure takes advantage of the periodic and predictable nature of V2X basic service messages. In particular, sensing-based SPS UEs reserve subchannels in the frequency domain for a random number of consecutive periodic transmissions in time domain. The number of slots for transmission and retransmissions within each periodic resource reservation period depends on the number of blind retransmissions (if any) and the resource selection procedure. The number of reserved subchannels per slot depends on the size of data to be transmitted.

#### 1) RESOURCE SELECTION PROCEDURE

The sensing-based resource selection procedure is composed of two stages: 1) a sensing procedure and 2) a resource selection procedure [39].

The sensing procedure is in charge of identifying the resources which are candidate for resource selection and is based on the decoding of the 1st-stage-SCI received from the surrounding UEs and on sidelink power measurements in terms of Reference Signal Received Power (RSRP) [33]. The sensing procedure is performed during the so-called *sensing window*, defined by the pre-configured parameter *T*_0_ and a UE-specific parameter *T*_proc,0_ that accounts for the time required to complete SCIs decoding and possibly perform measurements on DMRS for the sensing procedure. Specifically, if at time *n* the sensing-based resource selection is triggered, the UE will consider the sidelink measurements performed during the interval [*n* − *T*_0_, *n* − *T*_proc,0_). Sidelink RSRP measurements can be computed using the power spectral density of the signal received in the PSCCH or in the PSSCH, for which the UE has successfully decoded the 1st-stage-SCI. PSCCH RSRP and PSSCH RSRP are defined as the linear average over the power contributions (in Watts) of the resource elements that carry DMRS associated with PSCCH and PSSCH [43], respectively.

Based on the information extracted from the sensing, the resource selection procedure determines the resource(s) for sidelink transmissions [39]. For that, another window is defined, the *resource selection window*. The resource selection window is defined by the interval [*n* + *T*_1_, *n* + *T*_2_], where *T*_1_ and *T*_2_ are two parameters that are determined by the UE implementation [33]. *T*_2_ depends on the packet delay budget (PDB) and on an RRC pre-configured parameter called *T*_2,min_. In case PDB > *T*_2,min_, *T*_2_ is determined by the UE implementation and must meet the following condition: *T*_2_,min <= *T*_2_ <= PDB. In case PDB ≤ *T*_2_,min, *T*_2_ = PDB. *T*_1_ is selected so that *T*_proc,1_ <= *T*_1_, where *T*_proc,1_ is the time required to identify the candidate resources and select a subset of resources for sidelink transmission. The resource selection procedure is composed of two steps. First, the candidate resources within the resource selection window are identified. A resource is indicated as non-candidate if an SCI is received on that slot or the corresponding slot is reserved by a previous SCI, and the associated sidelink RSRP measurement is above a sidelink RSRP threshold [33]. The resulting set of candidate resources within the resource selection window should be at least a *X* % of the total resources within the resource selection window to proceed with the second step of the resource selection. The value of *X* is configured by RRC and can be 20 %, 35 % or 50 %. If this condition is not met, the RSRP threshold is increased by 3 dB and the procedure is repeated. Second, the transmitting UE performs the resource selection from the identified candidate resources (which may include initial transmissions and retransmissions). For that, a randomized resource selection from the identified candidate resources in the resource selection window is supported.

To exclude resources from the candidate pool based on sidelink measurements in previous slots, the resource reservation period (which is transmitted by the UEs in the 1st-stage-SCI) is introduced. As only the periodicity of transmissions can be extracted from the SCI, the UE that performs the resource selection uses this periodicity (if included in the decoded SCI) and assumes that the UE(s) that transmitted the SCI will do periodic transmissions with such a periodicity, during *Q* periods. This allows to identify and exclude the non-candidate resources of the resource selection window. According to [33], $Q=\left\lceil \frac{T_{\text{scal}}}{P_{\text{rsvp}}}\right\rceil$, where $P_{\text{rsvp}}$ refers to the resource reservation period decoded from the SCI, and $T_{\text{scal}}$ corresponds to *T*_2_ converted to units of ms [33].

As previously mentioned, NR V2X also supports a random resource selection [39]. In this case, the sensing procedure is omitted, and all the resources within the selection window that are part of the resource pool for sidelink are candidates for random selection.

**FIGURE 4.**
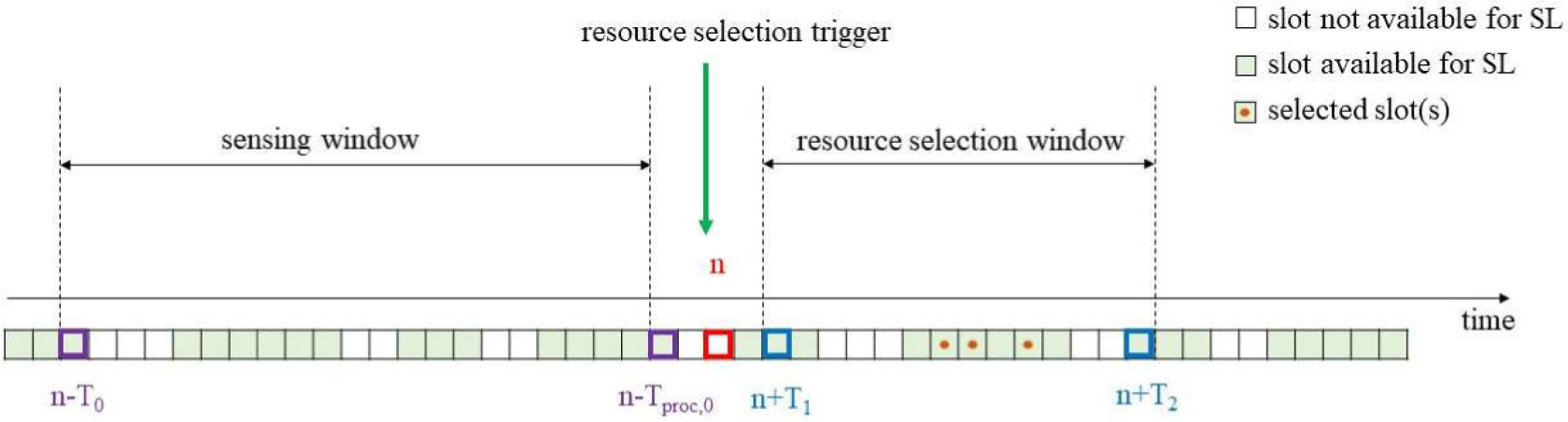
NR V2X Mode 2 resource selection procedure. Example with *T*_0_ = 20 slots, *T*_proc,0_ = 2 slots, *T*_1_ = 2 slots, and *T*_2_ = 16 slots.

Fig. 4 shows the resource selection procedure in NR V2X Mode 2. The figure illustrates the sensing window and resource selection window, with an example that uses *T*_0_ = 20 slots, *T*_proc,0_ = 2 slots, *T*_1_ = 2 slots, and *T*_2_ = 16 slots. Once the resource selection is triggered at time *n*, based on the measurements in the sensing window, the MAC scheduler determines the transmission resources within the resource selection window, which can be used for different MAC PDUs or to perform blind retransmissions.

#### 2) SEMI-PERSISTENT SCHEDULING

Once one or multiple resources are selected, the UE will consider periodic transmissions, using SPS. The transmission interval is defined by the Resource Reservation Period ($P_{\text{rsvp}}$), which is pre-configured by RRC and can take predefined values between 1 ms and 1000 ms [39]. $P_{\text{rsvp}}$ value is included in the 1st-stage-SCI, to allow other UEs to estimate which resources are reserved in the future based on SCI decoding. After using the resource for the number of transmissions equal to the Sidelink Resource Reselection Counter (SLRRC), a resource reselection is triggered. Whether to reselect or not, depends on the configured probability of keeping the current resources, hereafter referred as “probability of resource keep”. In particular, once SLRRC reaches zero, the UE either keeps the previous selection or selects new resources based on the pre-configured probability value. The value of SLRRC is randomly selected from the interval [5, 15] for $P_{\text{rsvp}}\;\geq \;100\;\text{ms}$. For $P_{\text{rsvp}}\;<\;100\;\text{ms}$, the value of SLRRC is randomly selected from the interval $\left\lceil 5\times \frac{100}{\max\left(20,P_{\text{rsvp}}\right)},15\times \frac{100}{\max\left(20,P_{\text{rsvp}}\right)}\right\rceil$ [39]. The standard also defines the maximum number of times that the same resource can be used for SPS through $C_{\text{resel}}=10\;\times \;\text{SLRRC}$, after which the resource reselection has to be triggered, independently of the probability of resource keep.

**FIGURE 5.**
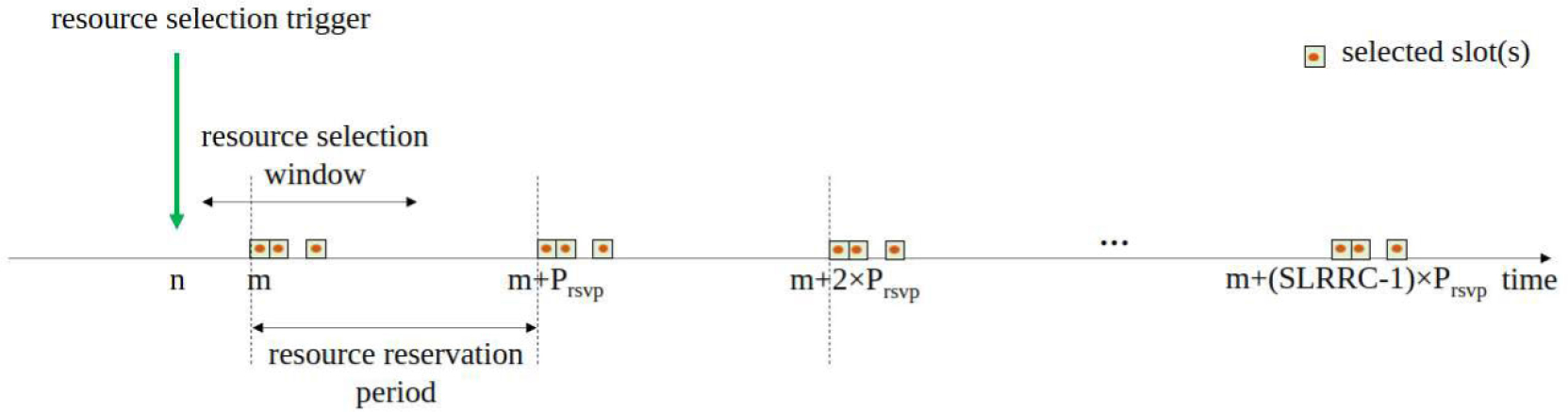
NR V2X Mode 2 semi-persistent scheduling.

An illustration of the SPS procedure for NR V2X Mode 2 is shown in Fig. 5. In the example, three resources are selected within the resource selection window (*m* in the figure is the slot index of the first selected resource), and these allocations are repeated every $P_{\text{rsvp}}$ for SLRRC times. Once the three transmissions in the interval starting at $m+\left(\text{SLRRC}-1\right)\times P_{\text{rsvp}}$ have been carried out, either the same selection is kept or a new resource selection procedure is triggered, based on the probability of resource keep.

## IV. NR V2X SIMULATION MODELS

This section describes the ns-3 based NR V2X simulator that we have built, as an extension of the NR 5G-LENA open source network simulator [25], and the V2X models that we have developed, including the design choices and implementation details.

**FIGURE 6.**
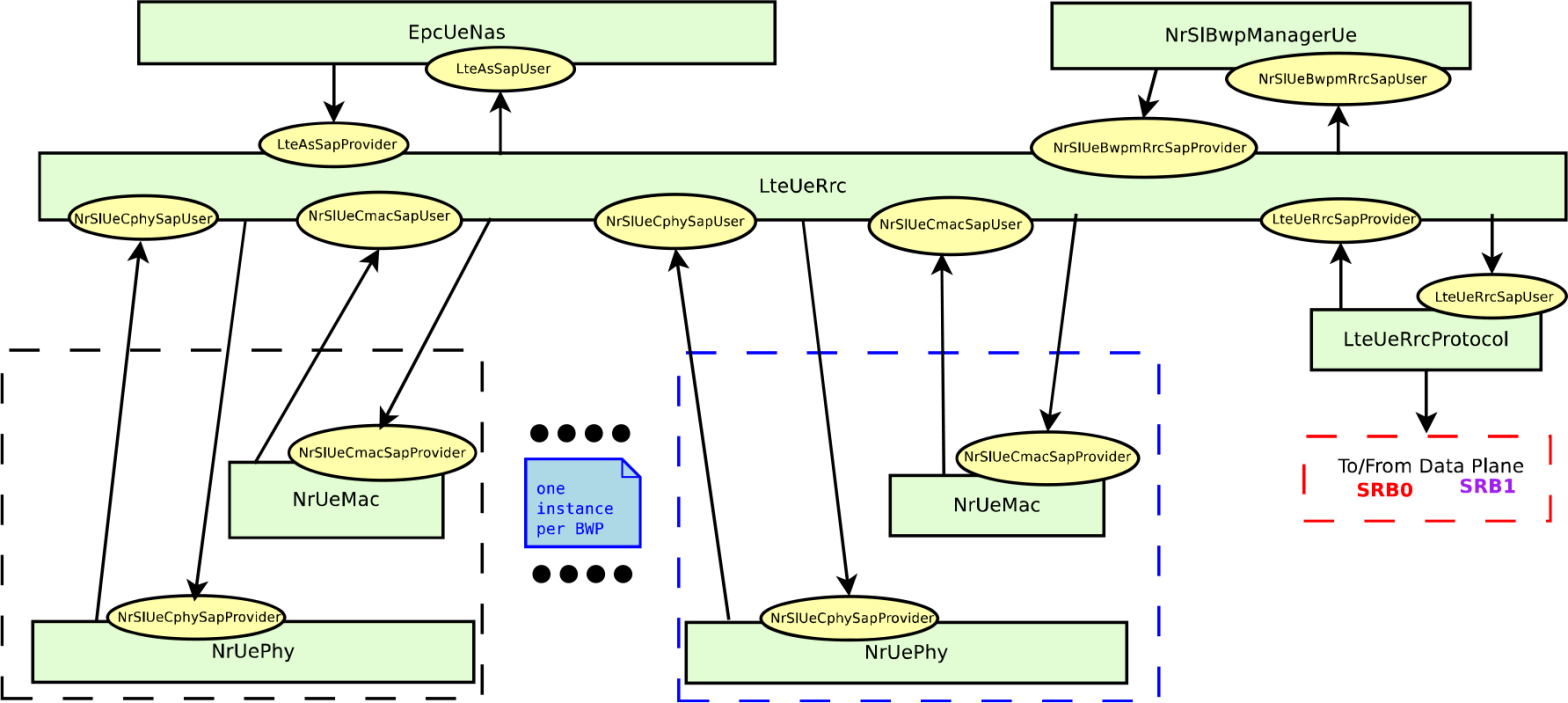
NR Sidelink UE control plane.

**FIGURE 7.**
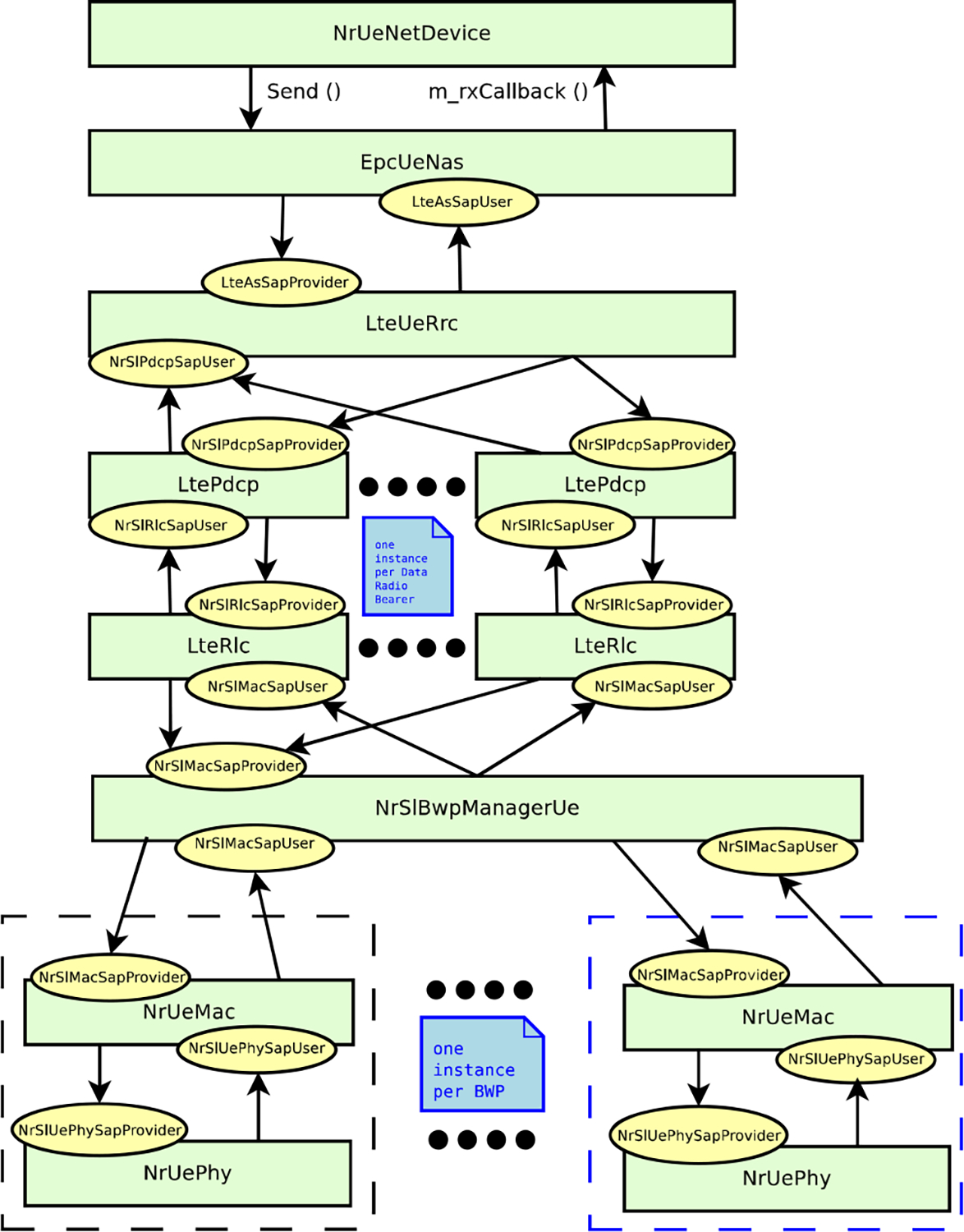
NR Sidelink UE data plane.

In our implementation, the data and control plane architectures of 5G-LENA UE nodes [25], shown in Fig. 7 and Fig. 6, have not been changed. However, the extension to support NR V2X functionalities involved some modifications at all layers of the protocol stack, from the Non-Access Stratum (NAS) and down to the PHY layer. One of the most important changes towards the implementation of NR V2X communications, compared to the typical cellular communications available in 5G-LENA simulator, is the introduction of sidelink, i.e., direct vehicle-to-vehicle communications. For that, the bearer establishment and RRC layer have been fully updated according to NR V2X RRC specification in TS 38.331 [35]. Also, MAC and PHY layers have been redesigned to implement NR V2X Mode 2 procedures using sensing-based SPS, as described in Section III-B, according to TS 38.321 for the MAC layer [39], and TS 38.211 and TS 38.212 for the PHY layer [32], [41]. In the simulator, as previously mentioned, we focus on NR V2X Mode 2 for out-of-coverage scenarios with broadcast communications and therefore, for the moment only blind retransmissions are considered.

Table 4 compares the main features of D2D, LTE C-V2X and NR V2X, as defined in the standard. Also, we compare the ns-3 based system level simulators available for D2D [20] and C-V2X [21], with the ns-3 NR V2X simulator presented in this paper. In Table 5, we detail the features and functionalities that are available in the developed NR V2X system-level simulator. The features listed for our simulator are those included in the first release, which allow the evaluation of a full NR V2X system with a subset of NR V2X features, but we plan to further progress with the module’s development to support more extensions and functionalities.

### A. NAS

The establishment and management of sessions occur at the highest layer on the control plane, the NAS. The current functionalities of the NAS layer involve establishment of Evolved Packet System (EPS) bearers, multiplexing uplink data packets coming from the upper layers into the appropriate EPS bearer by using the Traffic Flow Templates (TFTs) classifier. A TFT defines the rules for mapping IP packets to the right bearer based on IP addresses, ports, and type of service parameters.

For sidelink, the modifications are similar to the ones introduced in [20]. Specifically, NAS now supports the activation of sidelink bearers, mapping of Internet Protocol (IP) packets to the sidelink bearers based only on the IP destination address of the packets, and the transmission/reception of packets in NAS OFF state to support out-of-coverage scenarios.

### B. RRC

The RRC is the control plane protocol in charge of setting important parameters for the session. The modifications in the RRC include the creation of the sidelink bearers upon receiving a notification from NAS, and the pre-configuration of UEs in an out-of-coverage scenario. As mentioned earlier, the model currently focuses on the broadcast communication, therefore, as per the standard, it supports the creation of uni-directional sidelink radio bearers [48].

Regarding the UEs’ pre-configuration, the model implements all RRC Information Elements (IEs) needed to configure a UE [35]. This configuration is of key importance to perform sidelink communication when the gNB is absent. These IEs are mainly used for two purposes. The first is to configure the UE’s PHY layer parameters, e.g., numerology, symbols per slot, bandwidth, and TDD pattern. The second is to provide the sidelink resource pool(s) information to MAC and PHY layers. It is also worth mentioning that the model allows the configuration of multiple Bandwidth Parts (BWPs) for sidelink, where for each BWP, more than one resource pool can be configured through RRC. We note that, in spite of supporting multiple resource pools per BWP, only one pool could be active at one time [39]. Moreover, differently from the standard, which uses separate pools for transmission and reception [35], our model uses the same active pool for both.

### C. PDCP

The changes introduced in Packet Data Convergence Protocol (PDCP) layer are in line with LTE sidelink [20]. In particular, when it comes to sidelink, it is no longer possible to uniquely identify a logical channel only based on its Logical Channel Identifier (LCID). With sidelink communications, UEs independently assign the LCIDs to logical channels for each destination (i.e., Layer 2 group ID) to which they are transmitting. Thus, it is impossible for UEs to identify the packets if multiple transmitting UEs select the same LCID for the same group. To solve this, two more identifiers, i.e., source Layer-2 ID and destination Layer-2 ID, are included to identify the transmitting UE [44].

### D. RLC

The Radio Link Control (RLC) layer in the simulator already supports the so called Unacknowledged Mode (UM), which is the RLC mode used for sidelink broadcast communications. The only modifications made to the RLC layer are identical to the PDCP layer.

### E. MAC

The UE’s MAC layer has been extensively modified to transmit and receive sidelink transmissions. In the following, we explain these procedures in detail.

#### 1) MAC TRANSMITTING PROCEDURE

In out-of-coverage scenarios, UEs are required to perform the autonomous resource selection following Mode 2, which could be based on sensing-based or random selection procedures, as explained in Section III-B and Section III-A6, respectively. The first significant modification introduced in this respect is the new MAC scheduler interface. This interface allows the implementation of sidelink UE-specific schedulers, which could assign resources following specific strategies, e.g., fixed MCS, adaptive MCS based on CSI, etc. The UE MAC layer is extended to provide all the information needed by a scheduler to perform resource selection. For example, the information related to all the Logical Channels (LCs) of destinations the UE is interested in transmitting to, the total number of available subchannels, $N_{\max\_\text{reserve}}$, the maximum number of PSSCH transmissions, and most importantly, the RLC sidelink Buffer Status Reports (BSRs) of each LC that indicate how much sidelink traffic needs to be transmitted. The second important addition is the buffering of the sensing data reported by the UE’s PHY layer. This buffer behaves like a sensing window at the time of a sensing-based resource selection. It contains the sensing information for the interval [*n* − *T*_0_, *n* − *T*_proc,0_), where *n* is the slot at which the resource selection is triggered, and *T*_0_ is configured by the RRC while *T*_proc,0_ is a MAC layer parameter. In what follows we will dive into the details of UE’s MAC layer operation to perform sensing-based resource selection.

At slot *n*, when a resource selection is triggered for a destination, the MAC layer draws a random counter (SLRRC) based on the user configured $P_{\text{rsvp}}$ value, which is used to compute $C_{\text{resel}}$. Then, for an active pool configured by the RRC, it computes the candidate resources (i.e., available slots) for sidelink transmission based on the selection window parameters, *T*_1_, *T*_2,min_, and *T*_2_. Since the final resources must be selected based on sensing information, the MAC follows the procedure described in Section III-B1, to filter out the resources from the total available ones, which could be occupied by the other transmitting UEs. Once the filtered candidate resources’ list is ready, the MAC layer forwards it to the scheduler. Our model provides a sample scheduler, which as per the standard [35], randomly selects a number of slots, i.e., $N_{\text{selected}}$, for sidelink transmissions. The number of $N_{\text{selected}}$ slots depends on the number of slots that are available in the filtered list and the maximum number of configured PSSCH transmissions. If *K* denotes the total number of available slots, and $N_{\text{PSSCH,maxTx}}$ is the maximum number of PSSCH configured transmissions, then:

(1) $$N_{\text{selected}}=\{\begin{array}{ll} N_{\text{PSSCH},\max T\text{x}}, & \;\text{if}\;K\geq N_{\text{PSSCH},\max\text{Tx}}\\ K, & \text{otherwise} \end{array}$$

The sample scheduler uses a fixed MCS strategy.^2^ After selecting the $N_{\text{selected}}$ number of slots, the scheduler computes the maximum number of available contiguous subchannels, and using it as a maximum limit for the resources in the frequency domain, it derives the Transport Block Size (TBS) using the fixed MCS by taking into account the BSR of a LC, and the 5 bytes overhead of 2nd-stage-SCI, which needs to be multiplexed with data. Then, it randomly chooses the index of the starting subchannel for each slot. Finally, it prepares a sidelink allocation valid for the first resource reservation period deciding also aspects like which slots from the $N_{\text{selected}}$ have to carry the 1st-stage-SCI, the New Data Indicator (NDI), and the Redundancy Version (RV) number of each slot. The UE MAC layer, upon receiving this allocation, creates the SPS grants based on the configured value of $P_{\text{rsvp}}$ and the already drawn counters, i.e., SLRRC and $C_{\text{resel}}$. After using these grants for a number of transmissions equal to the SLRRC, a resource reselection is triggered. That is, once SLRRC reaches zero, the UE either keeps the previous selection or selects new resources based on the pre-configured probability of resource keep. Finally, if $C_{\text{resel}}$ reaches zero, the resource reselection is triggered, independently of this probability. As already discussed in Section III-A6, our model also supports a random resource selection. The only difference between the two approaches is that the random resource selection procedure does not filter the slots from the available ones before giving the list to the scheduler, so that the sensing information is not used.

Before forwarding the sidelink packets to the PHY layer, a check is performed at the beginning of each slot to ensure the availability of a valid grant for that slot. If there is, the MAC layer prepares two packet bursts, one for the 1st-stage-SCI and the second for the 2nd-stage-SCI plus data, and assigns a HARQ process ID to the data packet. It also saves this data packet into a HARQ buffer if blind retransmissions are configured. We note that the model allows to configure multiple (no limit for research purposes) sidelink/HARQ processes to allow continuous flow of data. After this, both packet bursts are forwarded to the lower layer. Upon receiving these packet bursts, the PHY, places them in a queue to be transmitted on the configured PSCCH and PSSCH symbols.

#### 2) MAC RECEIVING PROCEDURE

The UE’s MAC layer, upon receiving the PSSCH packet burst from the PHY, first retrieves the 2nd-stage-SCI to read the source Layer-2 ID and the destination Layer-2 ID of the received packet. As mentioned in section IV-C, these identifiers are used to map the received packet to its logical channel. If a bearer for the received packet is already established, the data packet is forwarded to the upper layers. Otherwise, the MAC asks the RRC to establish the bearer for the reception. Once this is done, the packet is forwarded to the upper layers.

### F. PHY

Similarly to the MAC layer, the PHY functionality can also be divided into transmitting and receiving procedures. In the following, we describe them in detail.

#### 1) PHY TRANSMITTING PROCEDURE

The 5G-LENA simulator accurately models (as per the standard) the numerology-dependent slot and OFDM symbol granularity. The state-machine of the PHY layer is mainly determined by the definition of the concept of start slot event and variable Transmission Time Interval (TTI) [26]. When the start slot event is triggered, the processing follows a logical order that involves the MAC and then the scheduler, before returning the control to the PHY. For sidelink, once the control gets back to the PHY, the PHY checks if the MAC has provided an allocation for the current slot. This allocation further consists of variable TTI allocations. The variable TTI means that the number of allocated symbols to physical sidelink channels (i.e., PSCCH and PSSCH) is variable, based on the sidelink configuration. Upon finding the allocation for the slot, the PHY layer transmits PSCCH and PSSCH PDUs on their respective symbols whose duration depend on the configured numerology and Cyclic Prefix (CP).

#### 2) PHY RECEIVING PROCEDURE

To receive the sidelink transmissions, one of the key enhancements of the PHY is the introduction of the ability to handle collisions/interference, also introduced in [20]. The interference model available in the 5G-LENA simulator was designed for a typical cellular communication. Its design assumes that a UE is interested in transmitting or receiving only from its serving gNB, and assumes no interference from the UEs served by the same gNB. Transmissions from other gNBs/UEs are simply considered as interference. In case of sidelink, especially in broadcast or groupcast, a UE is interested in transmitting to or receiving from multiple surrounding UEs. In this context, UEs in out-of-coverage scenarios or “UE-selected” mode can select the same (or overlapping) resources because the allocation is uncoordinated. Therefore, to determine which packet will be successfully decoded, the new implementation keeps track of the SINR values for each sidelink transmission.

As described earlier, currently our model supports the transmission and reception of timely multiplexed PSCCH and PSSCH. Thus, the PHY first receives signal(s) (i.e., 1st-stage-SCI) transmitted over PSCCH. This signal is used for two purposes: 1) to measure the RSRP required for the sensing-based resource selection, 2) to retrieve the information about the possible PSSCH transmission and retransmissions. The RSRP is computed using the 3 Resource Elements (REs) per RBs, carrying the 1st-stage-SCI, since the simulator does not explicitly include PSCCH DMRS. Moreover, for the sensing-based resource selection, the PHY measures the RSRP of each correctly decoded 1st-stage-SCI, from all the surrounding UEs. On the other hand, after computing the RSRP, if it is from the transmitter of interest, it reads the information encoded in the 1st-stage-SCI to receive the PSSCH transmission and its possible retransmissions.

Concerning the error model used for the reception of PSCCH and PSSCH transmission, we use the existing data plane error model in 5G-LENA [45], since the MCSs defined for PSSCH are the same as the ones defined for PDSCH/PUSCH. Also, we adopt such an error model for the PSCCH, using MCS0.

### G. CHANNEL MODELS

TR 37.885 [47] defines the system-level evaluation methodology for 5G V2X use cases, including the description and modeling of scenarios, deployment, mobility, antenna, traffic, and channel models. For channel modeling, TR 37.885 extends the geometry-based stochastic channel modeling framework introduced in TR 38.901 [49] for typical cellular communications, by adding the possibility to model wireless channel in vehicular environments and sidelink communications in which both the transmitter and the receiver are in motion. Two key scenarios are used for NR V2X evaluation [47]:

- Urban grid, which targets urban environments with a grid of buildings and roads with four lanes (two in each direction) between the buildings, and
- Highway, which targets highway environments with a highway composed of a total of six lanes, considering three lanes in each opposite direction.

For each scenario, TR 37.885 specifies new channel condition models, propagation models, and fast fading parameters capturing the characteristics of each environment.

The developed ns-3 NR V2X module includes the channel and antenna models for both V2X Urban grid and Highway scenarios, as defined in [47].

## V. NR V2X EVALUATION CAMPAIGNS

This section presents the simulation scenario that we have used to assess NR V2X performance. Then, we present multiple simulation campaigns and discuss the obtained end-to-end results.

### A. SCENARIO AND DEFINITION OF NEIGHBOR

**FIGURE 8.**
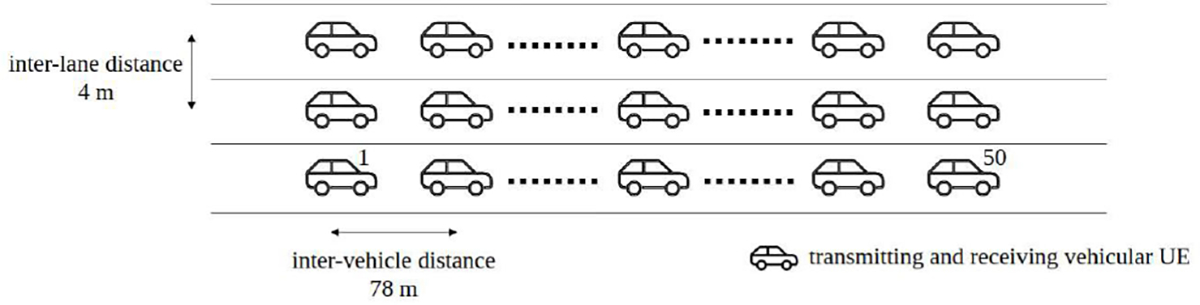
Highway scenario with 3 lanes and 50 vehicular UEs per lane moving at a speed of 140 km/h and spanning over 3.9 km.

We consider a V2X Highway scenario, as defined in 3GPP TR 37.885 [47]. The deployment is composed of multiple lanes in a 3.9 km highway road, with an inter-lane distance of 4 m. Within each lane, the inter-vehicle distance is 78 m, which is computed using the formula *max*(2, 2 × average speed m/s) defined in [47]. The UE dropping is implemented according to [47] Option A, in which all vehicles (100 %) are of Type 2 (i.e., passenger vehicle with an antenna height of 1.6 m), clustered dropping is not used, and the vehicle speed is set to 140 km/h in all the lanes. We consider 3 lanes with vehicles moving in the same direction, and 50 vehicles per lane. We focus on an out-of-coverage scenario, so that gNBs are disabled in the evaluation [47]. The considered deployment scenario is shown in Fig. 8.

We focus on a use case that targets the broadcast of basic service messages and by taking the inspiration from [50] we assume that all vehicular UEs are half duplex^3^ transceivers, which have the same packet size, generated at the same rate, and using a fixed MCS. Transmission is done over the 5.9 GHz band, assuming a channel bandwidth of 10 MHz [9]. The traffic model is characterized by periodic packet transmissions, with a packet size of 300 bytes, which are transmitted every 100 ms. This leads to a data rate of 24 kbit/s.

Moreover, in the considered scenario, each vehicular UE is a potential receiver. However, as per 3GPP [47], for the broadcast scenario, the Key Performance Indicators (KPIs), e.g., Packet Inter-reception Delay (PIR) and Packet Reception Ratio (PRR), for each UE must be computed by considering only those UEs that are located within a specific range of a certain distance from it, which is known as the “awareness range”. We consider an awareness range of 200 m, and we characterize as neighbors all those vehicular UEs located within such range from the source UE [50]. We also consider a throughput KPI, which according to its definition in 3GPP standard is computed without considering any awareness range, and is defined in the next subsection [47].

Table 6 reports the simulation parameters and functionalities, for NR V2X end-to-end evaluations. Through the simulation campaigns, we study the impact of specific NR V2X parameters, which are listed in Table 6 as variations of the baseline configuration (last column).

### B. SIMULATION CAMPAIGNS

The simulation campaigns are classified into two main blocks. Firstly, in Section V-C we study the impact of various NR V2X parameters on the performance of the sensing-based resource selection. In particular, we discuss a set of simulation campaigns, where we study the impact of the following parameters:

- NR V2X numerology (*μ*),
- NR V2X number of PSSCH transmissions of the same MAC PDU (including initial transmission and blind retransmissions) ($N_{\text{PSSCH,maxTx}}$),
- NR V2X Mode 2 selection window length (*T*_2_)
- NR V2X maximum number of resources per reservation ($N_{\max\_\text{reserve}}$),
- NR V2X Mode 2 probability of resource keep, and
- NR V2X MCS index for PSSCH.

Secondly, in Section V-D, we focus on comparing the performance of sensing-based and random resource selection procedures (both considered in 3GPP for NR V2X Mode 2). In this case, we consider a concrete system configuration, which corresponds to the baseline configuration shown in Table 6.

For each simulation campaign, 20 random channel realizations are performed, to get statistical significance. A single simulation has the duration of 10 simulated seconds. The constant bit rate applications start randomly within an interval of 100 ms, and run without interruption for 10 seconds.

As output statistics, we focus on the three KPIs defined for V2X evaluations in 3GPP [47], measured at the application layer:

- PIR: interval of time elapsed between two successful packet receptions of packets transmitted by a specific neighbouring UE. We consider the average PIR, averaging over the different successful receptions for each transmit-receive UE pair in the reception range. PIR is a range-based KPI, as per [47].
- PRR: for each packet transmitted by a UE, a ratio of the number of neighboring UEs that successfully receive that packet over the total number of neighboring UEs. We consider the average PRR, averaging over the different packets transmitted by a transmitting UE. PRR is a range-based KPI, as per [47].
- Throughput: total number of correctly received bytes over the simulation time, measured at the application layer, for each transmit-receive UE pair. As per [47], throughput is not range-based, so we consider all throughput values for those UEs that received some data bytes.

For each of the output statistics, we represent the Cumulative Distribution Function (CDF), over the different simulation runs. For each simulation campaign, we show three figures, one for each of the above mentioned output statistics, i.e., (a) PIR, (b) PRR, (c) throughput.

### C. SIMULATION RESULTS: SENSING-BASE RESOURCE ALLOCATION

#### 1) IMPACT OF NUMEROLOGY

**FIGURE 9.**
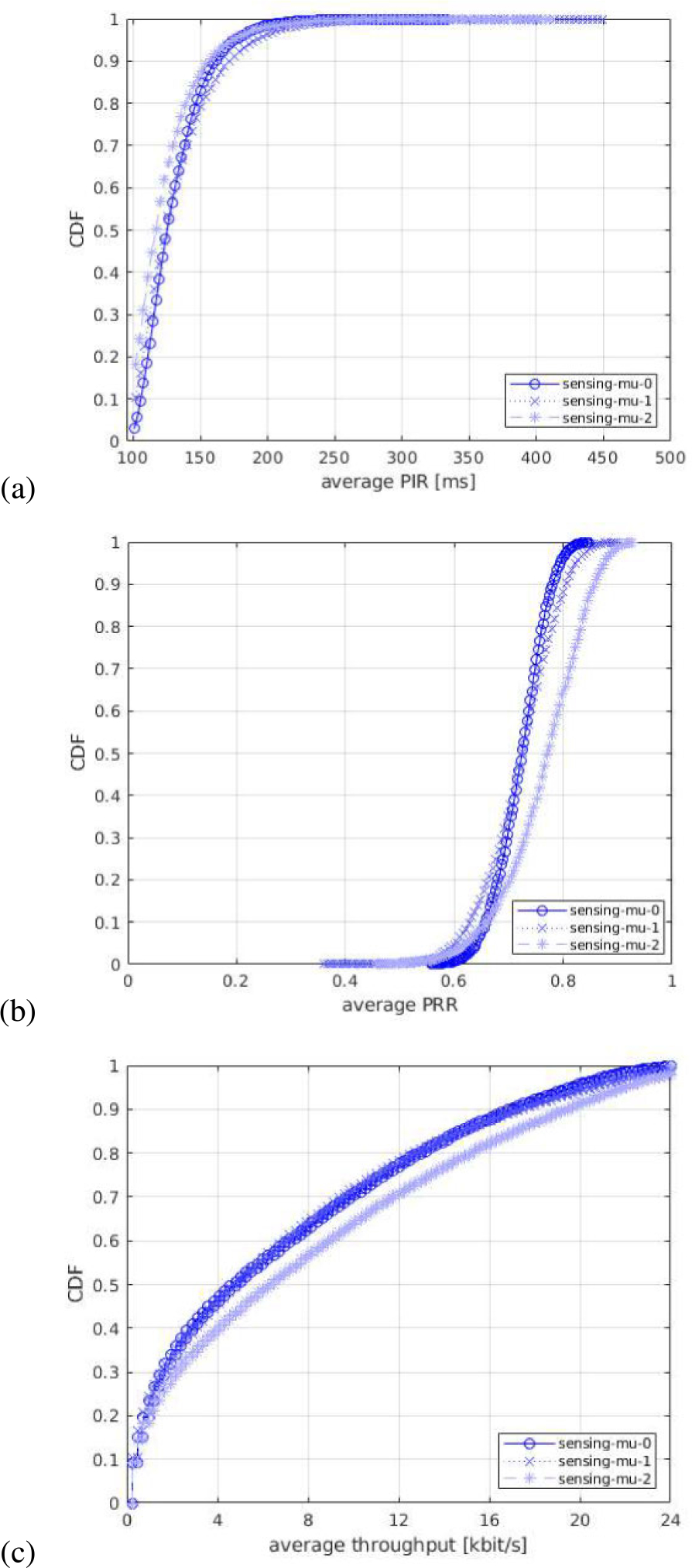
Impact of NR V2X numerology (*μ*). (a) PIR (ms), (b) PRR, (c) throughput (kbps).

In the first simulation campaign, we evaluate the impact of different NR numerologies. In these tests, we consider three different numerologies: *μ* = 0 (15 kHz SCS), *μ* = 1 (30 kHz SCS), and *μ* = 2 (60 kHz SCS), which are the three numerologies supported in NR standard for sub 6 GHz bands. Our comparison assumes that the same bandwidth is available for all the tested numerologies, which is also the common assumption in 3GPP evaluations. The numerologies are displayed in the legends of the figures as mu-0, mu-1, and mu-2, respectively. Fig. 9 shows the CDF statistics of the PIR, PRR, and throughput.

In Fig. 9.(a)–(b), we observe that the curves of PIR and PRR for the tested numerologies cross each other in different regions of the plot. The reason is a type of flexibility introduced by each numerology in terms of the number of available subchannels in the frequency domain, and different slot duration in the time domain. In NR, the processing times and the transmission durations are inversely proportional to the SCS, i.e., for a given bandwidth a lower SCS provides higher number of RBs while a higher SCS implies lower timings (i.e., shorter slot duration). For example, in our scenario with 10 MHz bandwidth and a subchannel size of 10 RBs, there are 5, 2, and 1 subchannel(s) available when using *μ* = 0, *μ* = 1, and *μ* = 2, respectively.^4^ Therefore, with the considered packet size of 300 bytes, which occupies only one subchannel^5^ a lower numerology, e.g., *μ* = 0 provides the maximum flexibility in the frequency domain, i.e., at a given time 5 UEs can occupy a single slot, which could reduce the collisions in the scenario. On the other hand, increasing the numerology, comes at the cost of a lower number of subchannels, so that we achieve a shorter slot length (as it is inversely proportional to the SCS), which makes that the resulting resource selection window length (set to 32 slots) results in a lower resource selection window in ms. For example, a 32 slots selection window results in 32 ms with *μ* = 0, 16 ms with *μ* = 1, and 8 ms with *μ* = 2. Interestingly, this reduction in terms of ms of the selection window, results in a reduced probability of overlapping of the resource selection windows of different transmitting UEs, because the resource reservation period is defined in ms and so it remains fixed independently of the numerology. This increases the gap in slots/time between the end of the selection window and the beginning of the following reservation period and consequently it helps reduce the probability of collisions between UE’s selection windows. However, when increasing the numerology from *μ* = 0 to *μ* = 1 we do reduce the number of available subchannels from 5 to 2 but we are also halving the selection window from 32 ms to 16 ms, which results in a very similar performance. In fact, having more subchannels with *μ* = 0 increases the PRR for some UEs thanks to the fact that UEs can better exploit diversity in frequency domain (see the tail of the PRR in Fig. 9.(b)). In this sense, we observe that for the tested scenario reducing the selection window length to half is not enough to achieve an appreciable performance gain. Differently, when the numerology is further increased to *μ* = 2, we can observe a performance gain achieved as a consequence of a lower selection window of 8 ms (i.e., 4times lower than *μ* = 0) as it is clearly shown in Fig. 9.(b)–(c). It is important to note that, even if the resource selection is based on sensing, the procedure requires that 20 % of the resources available for SL are candidates to perform a resource selection. If this requirement is not fulfilled, the RSRP threshold is increased by 3 dB until this condition is met. Because of that, collisions can still occur. Ultimately, the number of incorrect PSSCH receptions may imply packet losses, if they cannot be recovered by HARQ. This is effectively observed in the PRR and PIR statistics (see Fig. 9.(a)–(b)), for which, again, *μ* = 2 is observed to offer better performance, as it allows reduced packet collisions and a larger number of successfully decoded packets, as a consequence of the shorter slot duration.

All in all, the trade-off of having more subchannels with a lower numerology versus a lower resource selection window length with higher numerology results in comparable performance when using the three numerologies. However, using *μ* = 2 results in a lower slot duration and helps more UEs to achieve higher PRR, higher throughput, and lower PIR (which appears due to a lower probability of overlapping resource selection windows of different transmitting UEs) compared to *μ* = 0 and *μ* = 1.

#### 2) IMPACT OF NUMBER OF PSSCH TRANSMISSIONS

**FIGURE 10.**
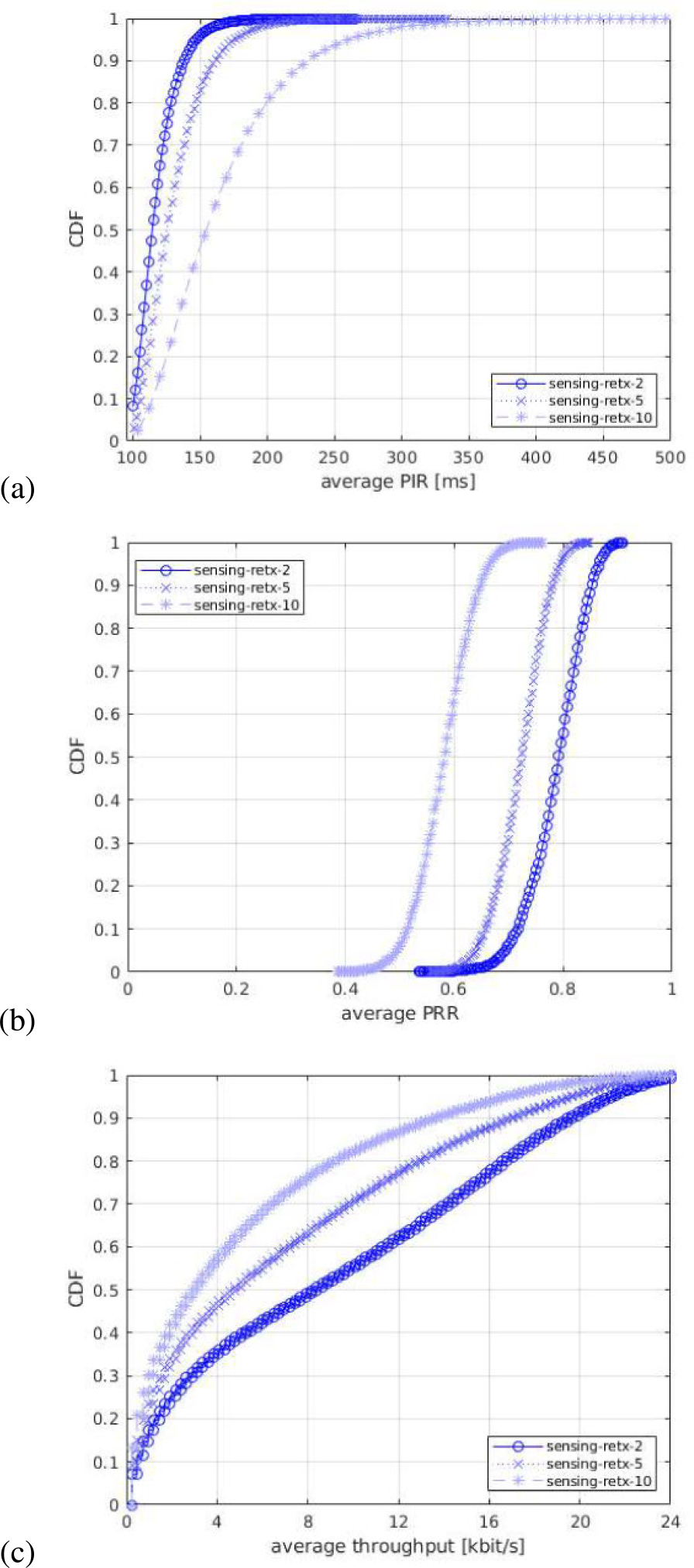
Impact of NR V2X number of PSSCH transmissions of the same MAC PDU ($N_{\text{PSSCH,maxTx}}$). (a) PIR (ms), (b) PRR, (c) throughput (kbps).

In the second simulation campaign, we assess the impact of using different numbers of PSSCH transmissions of the same MAC PDU. This parameter is also known as $N_{\text{PSSCH,maxTx}}$ and, in case of SPS with blind retransmissions, it corresponds to the maximum possible number of resources that can be selected by the resource selection procedure. In our tests, we use $N_{\text{PSSCH,maxTx}}\;=\;2,\;5,\;\text{and}\;10$. This includes one initial transmission and $N_{\text{PSSCH,maxTx}}\;-\;1$ blind retransmissions. They are displayed in the legends of the figures as retx-2, retx-5, and retx-10, respectively. Fig. 10 shows the CDF statistics of the PIR, PRR, and throughput.

As shown in Fig. 10, a lower number of PSSCH transmissions is beneficial in terms of all the performance indicators. The reason is that in the considered scenario, characterized by good propagation conditions, a lower $N_{\text{PSSCH,maxTx}}$ does not saturate the resources, which helps the sensing-based procedure at the transmitting UEs to fully exploit the flexibility in terms of the number of subchannels, i.e., 5 subchannels to choose from at each slot. After properly filtering the resources based on sensing, a lower $N_{\text{PSSCH,maxTx}}$, offers an improvement in PIR, PRR, and throughput compared to higher values of $N_{\text{PSSCH,maxTx}}$, which is due to the fact that the sensing procedure is properly avoiding collisions and a small number of PSSCH transmissions is enough to decode the packets because of the good propagation conditions. This definitely demonstrates the effectiveness of the sensing-based resource selection in vehicular scenarios.

In summary, in a scenario with good propagation conditions where it is likely that UEs are able to sense each other, a low number of PSSCH transmissions shows better performance for all the indicators when using sensing-based resource selection. The result changes when sensing is not activated. A similar campaign has also been conducted to study the impact of $N_{\text{PSSCH,maxTx}}$ for the non sensing case. The results are not shown here for the sake of brevity, but demonstrate that with a random resource selection, more PSSCH retransmissions of the same MAC PDU are needed to properly decode the packets and improve the throughput, PRR, and PIR performances.

#### 3) IMPACT OF RESOURCE SELECTION WINDOW LENGTH

**FIGURE 11.**
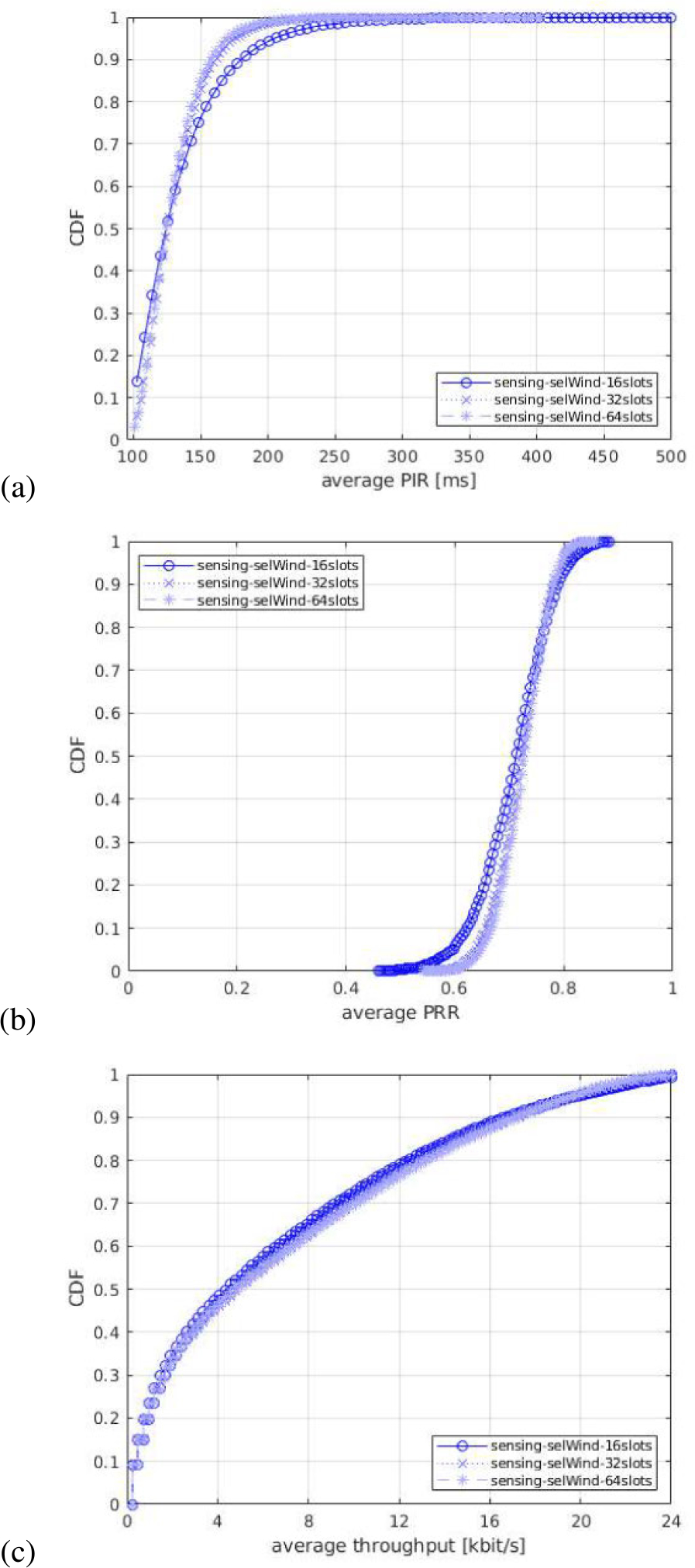
Impact of NR V2X selection window (*T*_2_−*T*_1_ + 1). (a) PIR (ms), (b) PRR, (c) throughput (kbps).

In the third simulation campaign, we evaluate the impact of different selection window lengths. To do so, we select *T*_1_ = 2 slots and vary *T*_2_ values. These two parameters, determine the start and the end point of the resource selection window. For *T*_2_, we consider three different values: *T*_2_ = 17 slots, *T*_2_ = 33 slots, and *T*_2_ = 65 slots, which result into a selection window length (*T*_2_−*T*_1_+1) of 16 slots, 32 slots, and 64 slots, respectively. Fig. 11 shows the results in terms of the PIR, PRR, and throughput.

In terms of PIR and PRR, the impact of different selection window length is not linear and different effects can be highlighted from the analysis of the results. On the one hand, a lower *T*_2_ causes PRR to decrease for almost 50% of the UEs, as observed in Fig. 11.(b), which is also reflected by higher PIR values in some cases in Fig. 11.(a). The reason is that a lower *T*_2_ generates more collisions due to the reduced number of slots in the resource selection procedure to select from. On the other hand, a higher *T*_2_ improves the performance, both in terms of PIR and PRR because a larger selection window provides more freedom to better randomize the resources among the various UEs. However, in some cases, the performance is very similar to what is observed with a lower *T*_2_. This is because by increasing the resource selection window length in ms, we also increase the probability of overlapping of the resource selection windows of different transmitting UEs, which ultimately results in more collisions. Due to the combination of these divergent effects, we observe that the curves of different *T*_2_ cross showing that in some cases a lower *T*_2_ can be beneficial, while in others it is not. In particular, we observe that high-PRR UEs benefit from *T*_2_ = 17 slots, while low-PRR UEs from *T*_2_ = 65 slots.

In general, a larger resource selection window shows benefits in terms of throughput, PIR, and PRR metrics, at the cost of a slight increase of the PIR of some UEs. As a result, it may be better to have more resources to select, even if for some specific cases, more collisions may appear because of the longer resource selection window length.

#### 4) IMPACT OF MAXIMUM NUMBER OF RESOURCES PER RESERVATION

**FIGURE 12.**
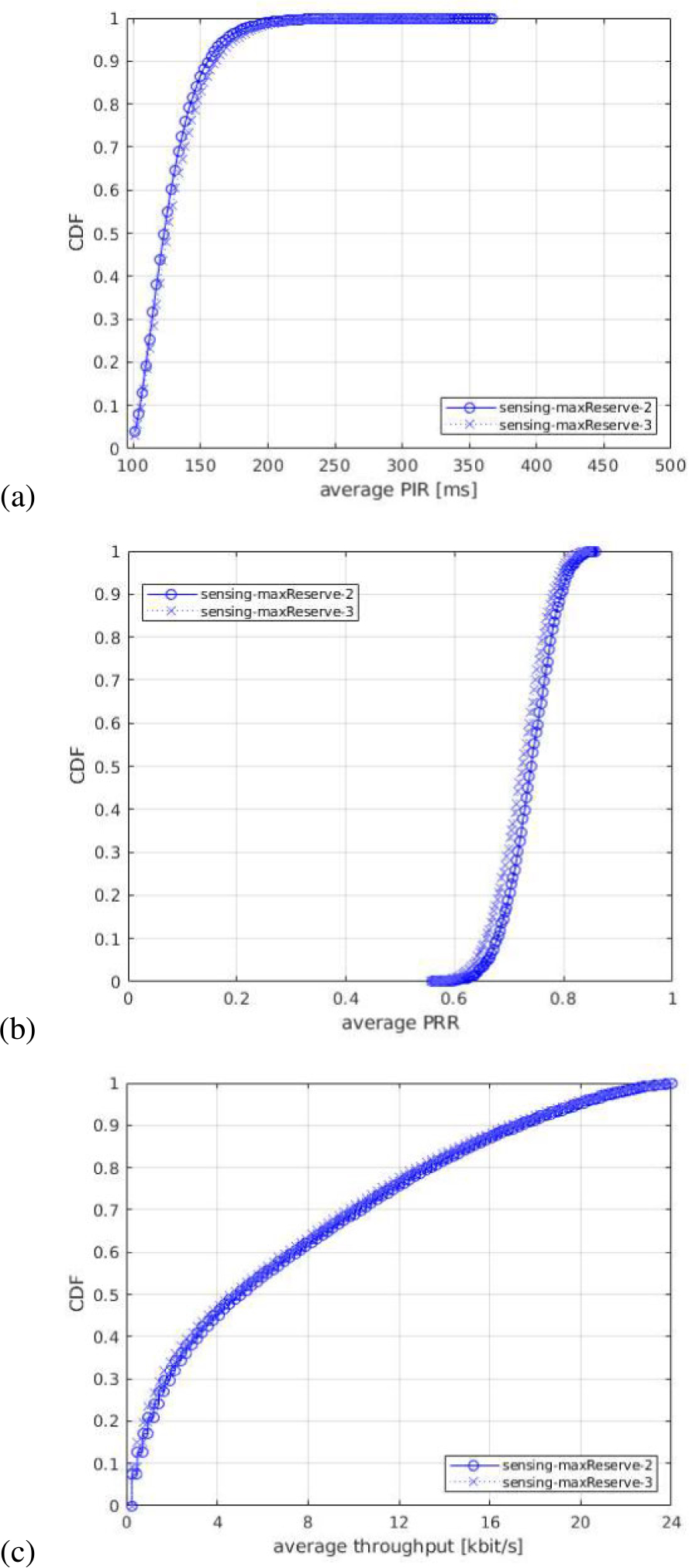
Impact of NR V2X maximum number of resources per reserve ($N_{\max\_\text{reserve}}$). (a) PIR (ms), (b) PRR, (c) throughput (kbps).

In the fourth simulation campaign, we vary the maximum number of resources per reservation ($N_{\max\_\text{reserve}}$). We consider the two values permitted in the NR V2X standard: $N_{\max\_\text{reserve}}\;=\;2$ and $N_{\max\_\text{reserve}}\;=\;3$. They are displayed in the legends of the figures as maxReserve-2 and maxReserve-3, respectively. Fig. 12 shows the results in terms of the PIR, PRR, and throughput.

The end-to-end performance obtained using the two values are very similar, but when considering $N_{\max\_\text{reserve}}\;=\;3$ the UEs experiences slightly higher PIR and slightly lower throughput, compared to $N_{\max\_\text{reserve}}\;=\;2$, as shown in Fig. 12.(a) and Fig. 12.(c), respectively. Also, the achieved PRR is higher with $N_{\max\_\text{reserve}}\;=\;2$ than with $N_{\max\_\text{reserve}}\;=\;3$ (see Fig. 12.(b)). The reason is that when there is a loss in the PSCCH channel, the UE has more opportunities to correctly receive and decode the 1st-stage-SCI using a retransmission with $N_{\max\_\text{reserve}}\;=\;2$, compared to the case of $N_{\max\_\text{reserve}}\;=\;3$, because with a lower $N_{\max\_\text{reserve}}$ there are more slots that carry 1st-stage-SCI messages in a resource reservation period. For example, in a simulation with $N_{\text{PSSCH,maxTx}}\;=\;5$ and $N_{\max\_\text{reserve}}\;=\;3$, there are only two 1st-stage-SCI messages transmitted in a resource reservation period (see Fig. 4), while with $N_{\max\_\text{reserve}}\;=\;2$, there are three 1st-stage-SCI messages. Specifically, if a UE fails to decode two 1st-stage-SCIs, it has no more opportunities to decode the data with $N_{\max\_\text{reserve}}\;=\;2$. However, in the same situation, with $N_{\max\_\text{reserve}}\;=\;2$, the receiving UE has another opportunity to decode the third 1st-stage-SCI and, potentially, the associated sidelink data. In addition, a larger $N_{\max\_\text{reserve}}$ value may cause the loss of sensing information because of the inherent characteristic of the sensing-based procedure, and, hence, more collisions. This is why, we observe a slightly better performance in terms of PIR, PRR, and throughput, when using $N_{\max\_\text{reserve}}\;=\;2$. However, let us note that said situation does not happen very often in our scenario, and that is why the difference between the performance is not much significant.

Consequently, the sensing-based resource selection is shown to be slightly more efficient when using a lower number of $N_{\max\_\text{reserve}}$, from all the metrics, because it allows for a more accurate sensing procedure. Particularly, it allows to detect more 1st-stage-SCIs from neighbor UEs, and so perform a better resource selection by excluding a larger set of slots that are being occupied by neighbor UEs. Also, for the receiving UEs, it provides more opportunities to decode the data.

#### 5) IMPACT OF PROBABILITY OF RESOURCE KEEP

**FIGURE 13.**
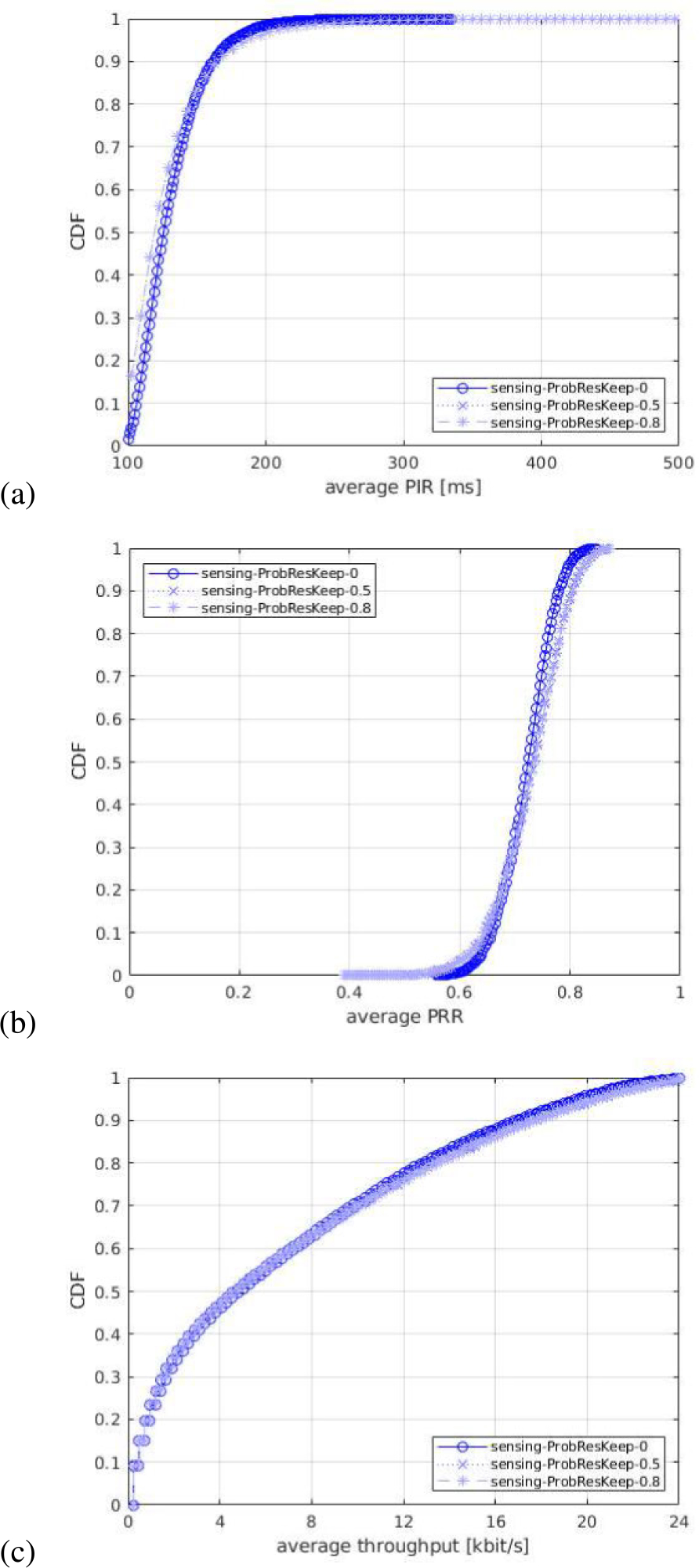
Impact of NR V2X probability of resource keep. (a) PIR (ms), (b) PRR, (c) throughput (kbps).

In the fifth simulation campaign, we study the impact of the probability of keeping the resources during reselection, by testing different values. As explained in Section 5, once SLRRC reaches zero, the UE either keeps the previous selection or selects new resources based on the pre-configured probability value. We consider three values, 0, 0.5, and 0.8. We note that, 0 and 0.8 are the standard minimum and the maximum values for the probability of keeping the resources [35]. They are identified in the legends of the figures as ProbResKeep-0, ProbResKeep-0.5, and ProbResKeep-0.8, respectively. Fig. 13 shows the results in terms of the PIR, PRR, and throughput. Let us note that for a transmitting UE, ProbResKeep-0 triggers the resource reselection procedure more often, as compared to ProbResKeep-0.5 and ProbResKeep-0.8.

Interestingly, we can see a trade-off in the obtained PIR and PRR performance when using different probabilities of keeping the resources. On the one hand, UEs that have correctly selected the resources (meaning their selection is not colliding with other UEs and its obtained KPIs are good enough) are benefited by not switching the selection, i.e., higher values of probability of resource keep are better. On the other hand, the UEs that did a wrong resource selection at the beginning (i.e., selected resources that may collide with other UEs’ selections) can benefit by reselecting the resources more often, i.e., by using lower values of probability of resource keep. For this reason, we see the crossing of the curves for PIR, PRR, and throughput curves.

#### 6) IMPACT OF MCS

**FIGURE 14.**
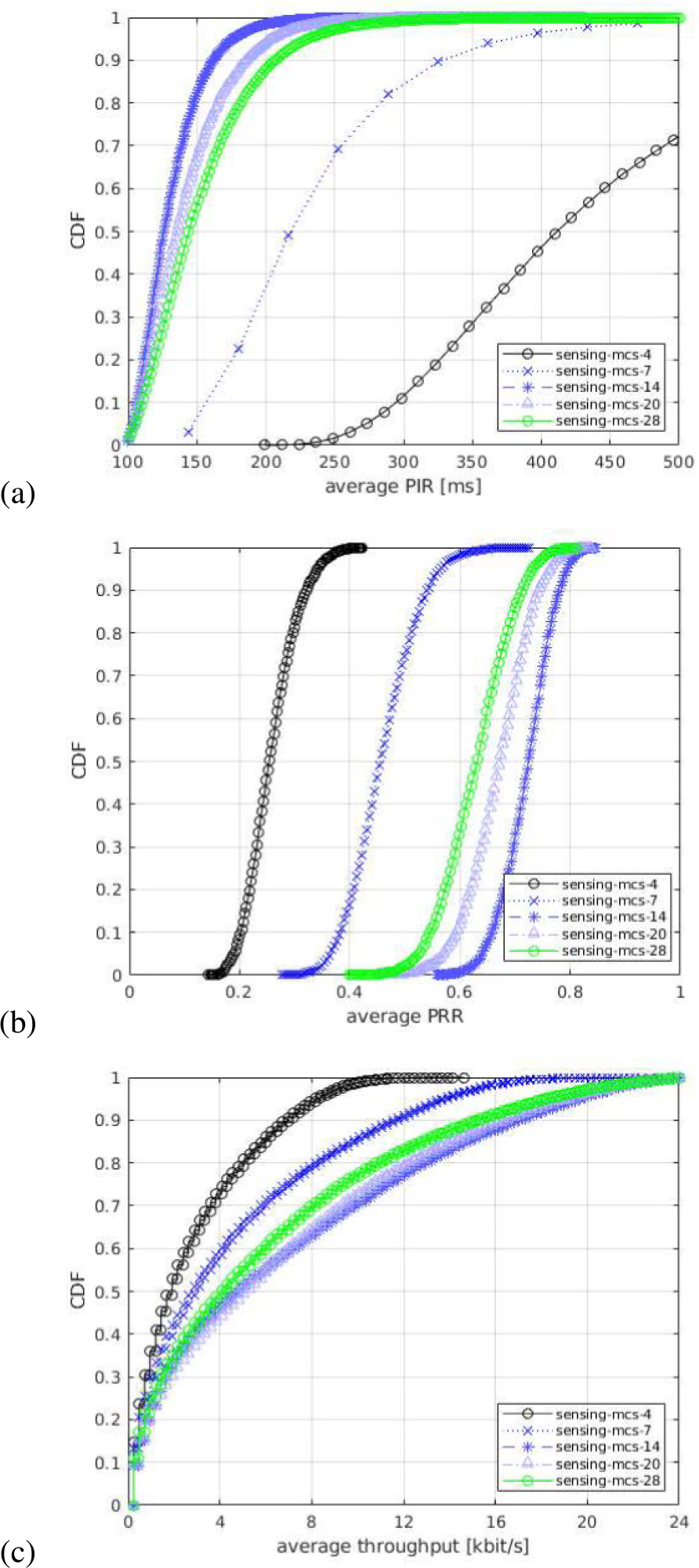
Impact of NR V2X MCS. (a) PIR (ms), (b) PRR, (c) throughput (kbps).

In the sixth simulation campaign, we evaluate the impact of different MCSs. We have used three MCS indexes adequate for V2X scenarios: MCS4, MCS7, and MCS14. Also, we include two more MCSs, just for comparison purposes: MCS20 and MCS28, even though, these two MCSs may be high for the considered reception range of 200 m. All the considered MCSs are displayed in the legends of the figures as mcs-4, mcs-7, mcs-14, mcs-20, and mcs-28, respectively. Fig. 14 shows the statistics of the PIR, PRR, and throughput.

Simulation results confirm that MCS14 is adequate for the considered simulation scenario with 200 m reception range. As expected, if a higher MCS is used (e.g., MCS20 and MCS28), the end-to-end performance in terms of PIR, PRR and throughput is degraded as compared to MCS14 (see Fig. 14.(a)–(c)). This is because a higher MCS is not suitable for large distances and leads to incorrect PHY receptions and packet losses that cannot be recovered even with HARQ combining. On the other hand, if an MCS lower than MCS14 is used, we also observe a performance degradation in all the considered KPIs (PIR, PRR, throughput), as shown in Fig. 14 for MCS4 and MCS7. In this case, even though lower MCS are more robust to packet losses and large distances, the reason of degradation is that the amount of data that fits in one subchannel gets reduced with a lower MCS, because of the reduced modulation order and the lower effective code rate. In particular, for the considered configuration and traffic pattern, with MCS14, one data packet can fit in one subchannel, as explained in Sec. V-C1. On the other hand, two subchannels are needed with MCS7 and three subchannels are required for MCS4. As more subchannels are needed with lower MCSs, the frequency diversity gain gets reduced. That is, less UEs can be multiplexed in frequency domain within the same slot, without interference. Accordingly, lower MCSs experience also PIR, PRR and throughput degradation as compared to MCS14.

All in all, an intermediate MCS (i.e., MCS14 in our case) is shown to be beneficial for V2X scenarios with a reception range of 200 m and the broadcast use cases, because it allows exploiting the frequency multiplexing gain and overcoming propagation losses, simultaneously.

#### D. SIMULATION RESULTS: SENSING-BASED VS RANDOM RESOURCE SELECTION

**FIGURE 15.**
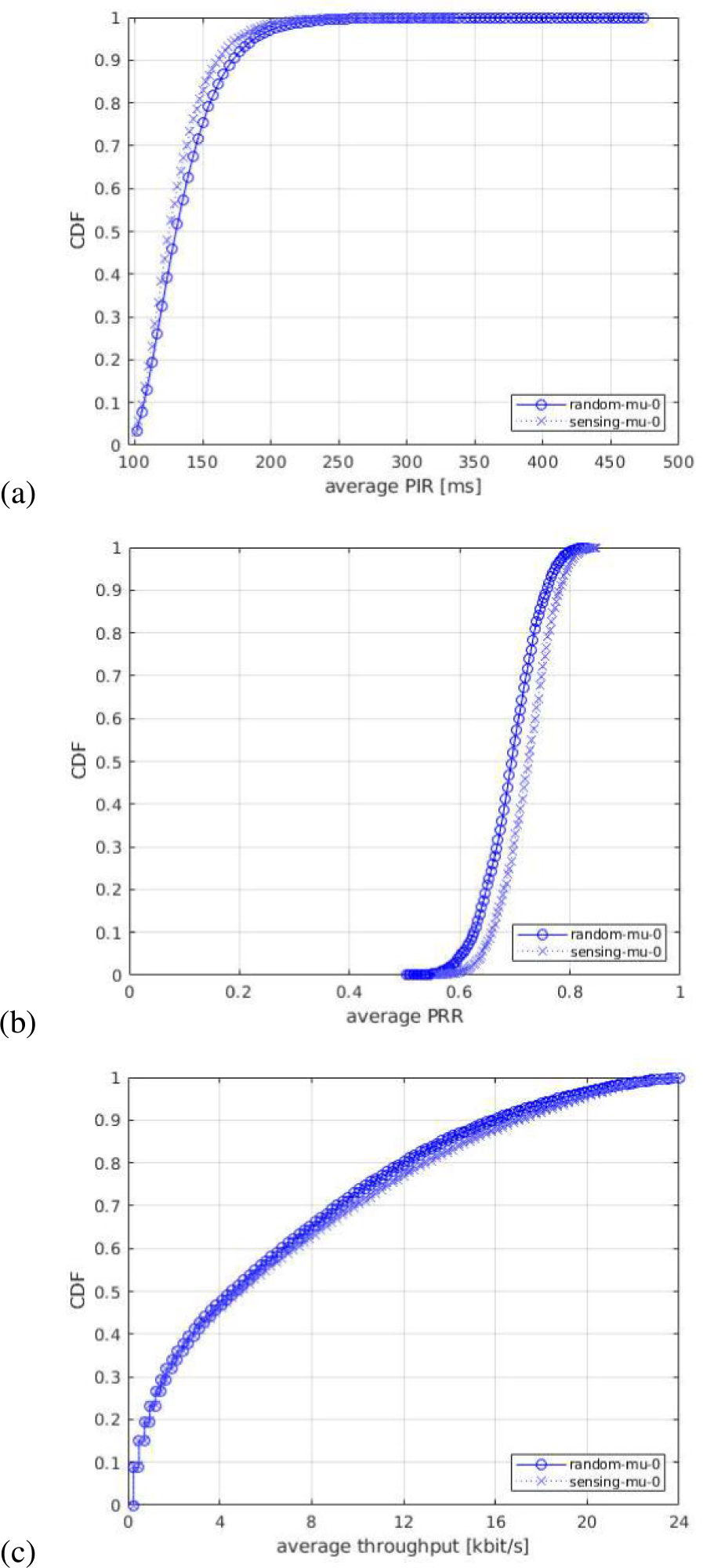
Impact of NR V2X resource selection procedure: sensing vs random. (a) PIR (ms), (b) PRR, (c) throughput (kbps).

In the last campaign, we consider the baseline configuration (i.e., numerology 0, number of PSSCH transmissions = 5, *T*_2_ = 32 slots, and $N_{\max\_\text{reserve}}\;=\;3$) and we focus on comparing sensing-based and random resource selection procedures for NR V2X. Notice that the sensing-based resource selection is defined by 3GPP Release 16, but Release 17 is also considering the random resource selection for power saving purposes, as previously discussed in Section III-B1. The two techniques are labeled in the legends of the figures as sensing and random, respectively. Fig. 15 shows the CDF of the PIR, PRR, and throughput.

Simulation results confirm that the sensing procedure ends up in a reduced PIR (see Fig. 15.(a)), an increased PRR (see Fig. 15.(b)), and a larger throughput (see Fig. 15.(c)). This is because sensing-based resource selection allows reducing the number of simultaneous PSSCH transmissions and incorrect PSSCH receptions in the reception range, as compared to the random resource selection procedure. As a consequence, due to the effectiveness of the sensing, we observe the PIR, PRR, and throughput improvement of sensing over the random selection procedure, in all the percentiles of the output statistics. So, these results confirm the expectations about the improvements given by sensing and show its performance gains in an end-to-end system-level simulator. The question that remains open though is whether the improvements provided by the sensing procedure are considerable enough to compensate for the increased complexity and power investment that sensing involves. The answer to this question is however out of the scope of this study and is left for a future work.

## VI. CONCLUSIONS

In this paper, we have presented an open-source, fullstack, end-to-end, standard-compliant network simulator for NR V2X, based on an extension of the already available ns-3 NR 5G-LENA simulator. We have started by reviewing the history of the different radio access technologies developed by 3GPP for sidelink communications and we have provided an exhaustive overview of NR V2X technology, currently under development in 3GPP, with special emphasis on NR V2X Mode 2 for autonomous resource selection, which is the main focus of this work. Successively, we have described with details the design and implementation of the developed simulator, which provides a useful and readable introduction to the module for a prospective and interested user. We have focused our work on broadcast communications for out-of-coverage scenarios, following the specifications of NR V2X Mode 2. For that, we have described the RRC pre-configuration and the NR V2X-compliant procedures at PHY and MAC layers, using UE autonomous resource selection based on sensing and semi-persistent scheduling.

Finally, we have presented a complete set of simulation campaigns, including the impact assessment of key NR V2X parameters, such as the numerology, the resource selection window, the number of retransmissions, the maximum number of resources per reservation, the probability of resource keep, and the MCS, as well as a comparison of sensing and random based resource selection procedures. The seven simulation campaigns that we have conducted have highlighted the following:

1. Only *μ* = 2 exhibits clear benefit to improve PIR, PRR, and throughput performance metrics.
2. A low number of blind retransmissions is already optimal in scenarios with good propagation conditions, because of the effectiveness of the sensing-based resource selection.
3. A trade-off is exhibited in terms of resource selection window length.
4. The maximum number of resources per reservation does not has an appreciable impact on the end-to-end performance.
5. A trade-off is observed in terms of the probability of resource keep for NR V2X with periodic traffic.
6. An intermediate MCS is shown to be optimal for V2X scenarios and broadcast use cases.
7. Appreciable but not significant gains are obtained by using sensing-based resource selection, in comparison to the random resource selection strategy, which is considered in 3GPP Release 17.

With these campaigns we have only touched the tip of the iceberg and many more studies can be conducted by the research community, considering the proposed open-source platform as a basis for analysis.

This work is only the first step to provide full support for end-to-end simulations of NR V2X networks, through an up-to-date and standard-compliant platform. Interesting future evaluations include higher transmitter densities, different scenarios, and different traffic models. In the near future we plan to extend the work to account for additional features and functionalities envisioned for NR V2X in Release 17 and onwards. Future works will include the feedback channel for HARQ-ACK and CSI reports, feedback-based retransmissions, new schedulers’ implementation, beam management procedures, and support for unicast communications. As the code is openly released, we hope that the developed simulator will contribute to research advancement in the areas of vehicular communications, public safety scenarios, and Beyond 5G, among others.

## Figures and Tables

**TABLE 1.** Evolution of sidelink in 3GPP (part 1: Release 12 till Release 15).

Release	WI/SI	Group	Objective	TR/TS
Release 12	SI: Study on LTE D2D Proximity Services - Radio Aspects	RAN1, RAN2, RAN3, RAN4	To define the methodology to evaluate LTE D2D proximity services, identify PHY layer options and enhancements	TR 36.843
Release 12	WI: Proximity-based Services	SA1, SA2, SA3	To specify service requirements for ProSe discovery and ProSe communication over E-UTRA	TS 21.905, 22.115, 22.278, 23.002, 23.122, 23.303, 23.401, 23.402, 24.301, 33.220, 33.303, 33.833, 36.413, 36.423
Release 13	WI: Enhanced LTE D2D Proximity Services	RAN2, RAN1, RAN3, RAN4	To define enhancements to LTE D2D communications and discovery meeting requirements for public safety applications	TS 36.101, 36.104, 36.133, 36.141, 36.211, 36.213, 36.214, 36.300, 36.301, 36.304, 36.306, 36.321 36.331, 36.413, 36.423
Release 13	WI: Enhancements to Proximity-based Services	SA1	To support stage 2/3 development during Release 13 and support end of Release 12 maintenance to review and ensure that Release 13 TS 22.278 and TS 22.115 contain all agreed ProSe Stage 1 requirements	TS 23.303, 33.303
Release 14	SI: Study on LTE support for V2X services	SA1	To study service requirements for V2P, V2P, V2N/V2I	TR 22.885
Release 14	WI: LTE support for V2X services	SA1	To specify service requirements for V2P, V2P, V2N/V2I	TS 22.185
Release 14	WI: Support for V2V services based on LTE sidelink	RAN1, RAN2, RAN3, RAN4	To specify LTE sidelink enhancements for V2V services defined in TR 22.885	TS 36.101, 36.104, 36.133, 36.141, 36.201, 36.211, 36.212, 36.213, 36.214, 36.300, 36.302, 36.304, 36.306, 36.307, 36.321, 36.323, 36.331, 36.413, 36.423
Release 15	WI: ProSe Support for Band 72 in LTE	RAN5	To update the 3GPP RAN WG5 RF, RRM and Protocol conformance test specification with the support of ProSe for Band 72	TS 36.508, 36.521
Release 15	WI: Remote UE access via relay UE	SA1	To specify service requirements for a UE with UICC to connect with network via an Evolved ProSe UE-to-Network Relay	TS 22.011, 22.115, 22.278,
Release 15	SI: Study on Enhancement of 3GPP support for V2X services	SA1	To identify use cases and potential service requirements to enhance 3GPP support for V2X service in safety and non-safety V2X scenarios	TR 22.886
Release 15	WI: Enhancement of 3GPP support for V2X scenarios	SA1	To specify service requirements to enhance 3GPP support for V2X scenarios valid for the 3GPP systems (i.e., 5G, EPS), including the transport layer support for safety and non-safety V2X scenarios	TS 22.186
Release 15	WI: V2X new band combinations for LTE	RAN4	To specify RAN4 RF requirements for the concurrent operation of additional LTE Uu frequency bands and PC5 operation on Band 47 and for concurrent operation of LTE Uu Carrier Aggregation and PC5 operation on Band 47	TS 36.101, 36.307
Release 15	WI: Enhancements on LTE-based V2X services	RAN1, RAN2, RAN3	To define enhancements on LTE-based V2X Services	TS 23.285, 23.303, 24.334, 24.385, 24.386, 36.101, 36.133, 36.201, 36.211, 36.212, 36.213, 36.300, 36.302, 36.304, 36.306, 36.321, 36.323, 36.331
Release 15	SI: Study on security aspects for LTE support of V2X services	SA2	To identify and evaluate potential architecture enhancements needed to operate LTE-based V2X (V2V, V2I/N, and V2P), based on vehicular services requirements defined in SA1 V2X LTE and determine which of the solutions can proceed to normative specification	TR 33.885
Release 15	SI: Study on evaluation methodology of new V2X use cases for LTE and NR	RAN1	To establish the evaluation methodology to evaluate technical solutions supporting the full set of 5G V2X use cases as identified in TR 22.886 and the full set of 5G RAN requirements in TR 38.913	TR 37.885
Release 15	SI: Study on further enhancements to LTE Device to Device (D2D), UE to network relays for IoT (Internet of Things) and wearables	RAN2, RAN1, RAN3, RAN4	To study enhancements to Prose UE-to-network relaying and to the LTE D2D framework for commercial and public safety applications such as wearable devices	TR 36.746

**TABLE 2.** Evolution of sidelink in 3GPP (part 2: Release 16 till Release 17).

Release	WI/SI	Group	Objective	TR/TS
Release 16	SI: Study on Improvement of V2X Service Handling	SA1	To identify use cases and potential service requirements to enhance 3GPP support for V2X	TR 22.886
Release 16	WI: Improvement of V2X Service Handling	SA1	To define use cases and potential service requirements to enhance 3GPP support for V2X, based on the studies in TR 22.886	TS 22.186
Release 16	SI: Study on application layer support for V2X services	SA6	To develop key issues, corresponding architecture requirements and solution recommendations to enable the application layer support for V2X services over 3GPP systems	TR 23.795
Release 16	WI: Application layer support for V2X services	SA6	To define architecture requirements, functional architecture, procedure and information flows, based on solutions and conclusions reached in TR 23.795	TS 23.286, 23.795, 24.486, 24.587, 27.007, 29.486
Release 16	SI: Study on architecture enhancements for the Evolved Packet System (EPS) and the 5G System (5GS) to support advanced V2X services	SA2	To identify and evaluate potential architecture enhancements of EPS and 5G System design needed to support advanced V2X services identified in TR 22.886	TR 23.786
Release 16	WI: Architecture enhancements for 3GPP support of advanced V2X services	SA2	To specify architecture enhancements of 5G system to support advanced V2X services as per conclusions reached within TR 23.786	TS 23.008, 23.122, 23.285, 23.287, 23.501, 23.502, 23.503, 24.007, 24.301, 24.385, 24.386, 24.501, 24.587, 24.588, 27.007, 29.122, 29.230, 29.272, 29.274, 29.388, 29.502, 29.503, 29.504, 29.505, 29.510, 29.512, 29.513, 29.514, 29.518, 29.519, 29.520, 29.522, 29.525, 29.571, 31.102, 33.185, 33.535, 33.536, 38.413, TS 38.423
Release 16	SI: Study on NR Vehicle-to-Every thing (V2X)	RAN1, RAN2, RAN3	To study sidelink design, Uu enhancements for advanced V2X use cases, Uu-based sidelink resource allocation/configuration, RAT/Interface selection for operation, QoS management, and coexistence	TR 38.885
Release 16	SI: Study on V2X Media Handling and Interaction	SA4	To study use cases relevant to transmission of multimedia over 3GPP and detail the requirements and procedures for media capturing, compression, and transmission	TR 26.985
Release 16	SI: Study on Security Aspects of 3GPP support for Advanced V2X Services	SA3	To provide security and privacy analysis of eV2X system architecture, derive potential security and privacy requirements, and evaluate security and privacy solutions for protection of it	TR 33.836
Release 16	WI: 5G V2X with NR sidelink	RAN1, RAN2, RAN3, RAN4	To specify radio solutions that are necessary for NR to support advanced V2X services (except the remote driving use case which was studied in TR 38.824) based on the study outcome captured in TR 38.885	TS 36.133, 36.300, 36.304, 36.306, 36.321, 36.331, 36.413, 36.423, 37.324, 37.340, 38.101, 38.104, 38.133, 38.201, 38.202, 38.211, 38.212, 38.213, 38.214, 38.215, 38.300, 38.304, 38.306, 38.321, 38.323, 38.331, 38.413, 38.423, 38.460, 38.463, 38.470, 38.473, 38.886
Release 17	WI: NR Sidelink enhancement	RAN1, RAN2, RAN4	To specify radio solutions that can enhance NR sidelink for the V2X, public safety and commercial use cases, with special focus on power saving, enhanced reliability and reduced latency	[none yet]
Release 17	SI: Study on NR Sidelink relay	RAN2	To study single-hop NR sidelink-based relay	TR 38.836
Release 17	SI: Study on enhancements to application layer support for V2X services	SA6	To study enhancements to the application architecture to support V2X services specified in 3GPP TS 23.286	TR 23.764
Release 17	WI: Enhanced application layer support for V2X services	SA6	To define enhancements to the application architecture to support V2X services specified in 3GPP TS 23.286	TS 23.286, 23.434, 27.007
Release 17	WI: Band combinations for concurrent operation of NR/LTE Uu bands/band combinations and one NR/LTE V2X PC5 band	RAN4	To specify band specific RF requirements for the concurrent operation of NR Uu and NR PC5, LTE Uu and NR PC5, NR Uu and LTE PC5	TR 37.875, TS 38.101
Release 17	SI: Study on V2X services - Phase 2	SA2	To study procedures for V2X authorization and V2X communication	TR 23.776

**TABLE 3.** Sub 6 GHz NR V2X bands.

V2X operating bands	Sidelink (SL) Transmission operating band *F*_UL_low_ - *F*_UL_high_ [MHz]	Sidelink (SL) Reception operating band *F*_DL_low_ - *F*_DL_high_ [MHz]	Duplex Mode	Sub carrier spacing [kHz]	Supported bandwidth [MHz]
n38 (Licensed)	2570 – 2620	2570 – 2620	TDD	15, 30, 60	10, 20, 30, 40
n47 (Unlicensed)	5855 – 5925	5855 – 5925	TDD	15, 30, 60	10, 20, 30, 40

**TABLE 4.** Comparison of D2D, LTE C-V2X, and NR V2X in the standard and ns-3.

	D2D standard	D2D ns-3 [20]	LTE C-V2X standard	C-V2X ns-3 [21]	NR Y2X standard	NR V2X ns-3 [this paper]
Communication types	groupcast	broadcast	broadcast	broadcast	broadcast, groupcast, unicast	broadcast
MCS	QPSK, 16QAM	QPSK, 16QAM	QPSK, 16QAM, 64QAM	QPSK, 16QAM, 64QAM	QPSK, 16QAM, 64QAM, 256QAM	QPSK, 16QAM, 64QAM, 256QAM
Waveform	SC-FDMA	SC-FDMA	SC-FDMA	SC-FDMA	OFDMA	OFDMA
Frequency range	sub 6 GHz	sub 6 GHz	sub 6 GHz	sub 6 GHz	sub-6 GHz, mmWave	sub-6 GHz, mmWave
Subcarrier spacing	15 kHz	15 kHz	15 kHz	15 kHz	sub-6 GHz: 15, 30, 60 kHz, mmWave: 60, 120 kHz	sub-6 GHz: 15, 30, 60 kHz, mmWave: 60, 120 kHz
Duplexing modes	FDD, TDD	FDD	FDD, TDD	FDD	FDD, TDD	TDD
Retransmissions	blind	blind	blind	blind	broadcast: blind, groupcast: blind, feedback-based, unicast: blind, feedback-based	broadcast: blind
PHY channels	PSCCH, PSSCH, PSDCH, PSBCH	PSCCH, PSSCH, PSDCH, PSBCH	PSCCH, PSSCH, PSBCH	PSCCH, PSSCH, PSBCH	PSCCH, PSSCH, PSBCH, PSFCH	PSCCH, PSSCH
Control and data multiplexing	frequency, time	time	frequency	frequency	frequency, time	time
Scheduling interval	1 subframe	1 subframe	1 subframe	1 subframe	1 slot	1 slot
Sidelink modes	1 and 2	1 and 2	3 and 4	4	1 and 2	2

**TABLE 5.** NR V2X models.

	NR V2X
Frame structure	TDD NR-compliant frame structure with slots and OFDM symbols of numerology-dependent length [25], [44]: - frame: 10 ms, subframe: 1 ms - each subframe has 2*^μ^* slots (associated to 15 × 2*^μ^* kHz SCS) - numerologies *μ*=0, 1, 2, 3, 4 are supported - each slot is composed of 14 OFDM symbols Support for multiple bandwidth parts [25]: - more than one BWP can be configured for sidelink - each BWP can have pre-configured multiple sidelink resource pools, but only one pool can be active at a time
Duplexing mode	TDD - the TDD pattern is flexible in length and composition, and can include downlink-only slots, uplink-only slots, or flexible slots (in which downlink and uplink transmissions can occur). An example of TDD pattern is [D F U U U].
Sidelink resource pool	- sidelink transmission is only allowed in uplink-only slots, and whether an uplink slot is available for sidelink or not is specified through the SL bitmap. An example of the SL bitmap is [1 1 1 1 1 1 0 0 0]. - within the uplink slots available for sidelink, the symbols available for sidelink are RRC pre-configured and our default structure is as follows: PSCCH can occupy 1 or 2 starting symbols, and depending on the PSCCH allocation, 2nd to 13th or 3rd to 13th symbols are available for PSSCH, and the 14th symbol is left empty as a guard period - in frequency domain, RRC pre-configures the subchannel size (in number of RBs), and as per this configured size, divides the available bandwidth in number of available subchannels.
SL data/control channels	- PSSCH and PSCCH are multiplexed in time - PSSCH and PSCCH are sent and received quasi-omnidirectionally at the UEs
Error models	NR PHY abstraction for PSSCH and PSCCH channels [45] including support for MCS Table1 and Table2 [33], MCS LDPC coding and block segmentation [41]
Modulation	OFDM
Channel Coding	LDPC
MCS	QPSK, 16-QAM, 64-QAM, 256-QAM
HARQ	NR PHY abstraction for HARQ includes support for HARQ-IR and HARQ-CC
Retransmissions	Blind retransmissions, including up to a pre-configured number with retransmission combining
Resource allocation	sensing-based and random resource selections are supported
Link adaptation	Fixed MCS
Antenna models	3GPP-compliant [46]: - Antenna arrays: 1 uniform planar array per UE, *M* × *N* antenna elements, no polarization - Antenna elements: isotropical and directional radiation are supported
Channel models	3GPP-compliant [47], supporting Urban grid and Highway scenarios, in both sub 6 GHz and mm Wave bands

**TABLE 6.** Main scenario simulation parameters (baseline configuration and its variations).

Parameter	Value (baseline)	Value (variations)

**Deployment and propagation parameters:**		

Channel model	3GPP Highway	
Deployment	3 lanes, 5 vehicles per lane	
Carrier frequency	5.89 GHz	
Channel bandwidth	10 MHz	
Noise power spectral density	-174 dBm/Hz	
UE antenna height	1.6 m	
UE speed	140 km/h	

**Traffic parameters:**		

Application packet size	300 Bytes	
Inter-packet arrival time	100 ms	
Application load	24 kbit/s	

**Device parameters:**		

UE antennas	uniform planar array 1×2	
UE transmit power	23 dBm	
UE noise figure	5dB	

**NR V2X parameters and functionalities:**		

Frame structure	*μ*=0 (SCS=15 kHz)	*μ*=1 (SCS=30 kHz), *μ*=2 (SCS=60 kHz)
TDD pattern	[D D D F U U U U U U]	
Sidelink bitmap	[1 1 1 1 1 1 0 0 0 1 1 1]	
Subchannel size (*N*)	10 RBs	
PSCCH symbols	1	
PSSCH symbols	12	
Link adaptation	fixed MCS	
MCS PSSCH	MCS 14 (MCS Table1)	MCS 4, MCS 7, MCS 20, MCS 28
MCS PSCCH	MCS 0 (MCS Table1)	
Error model	NR PHY abstraction based on EESM [45] for PSSCH and PSCCH	
Number of PSSCH transmissions ($N_{\text{PSSCH,maxTx}}$)	5	2, 10
HARQ combining method	HARQ incremental redundancy	
MAC resource selection	sensing-based resource selection	random resource selection
RLC mode	RLC-UM	
RLC buffer size	999999999 Bytes	

**NR V2X Mode 2 parameters:**		

Sensing window (*T*_0_)	100 ms	
*T*_2_	33 slots	17 slots, 65 slots
*T*_1_	2 slots	
*T*_proc,0_	1 slot	
Percentage of resources must be selected in a selection window	20 %	
Max num per reserve ($N_{\max\_\text{reserve}}$)	3	2
Probability of resource keep	0	0.5, 0.8
Resource reservation period ($P_{\text{rsvp}}$)	100 ms	
RSRP threshold	-128 dBm	

## Footnotes

1 The fexible slot is used to provide the necessary guard time for downlink to uplink switching in TDD systems.

2 Note that adaptive MCS strategy makes sense in unicast and groupcast communications when the CSI from the receiving UE can be acquired through the PSSCH. As in this paper we have focused on broadcast communications, without PSFCH, the fixed MCS strategy is the adequate one. However, the implemented scheduler interface is general enough to accommodate more sophisticated schedulers.

3 In this paper, we use sidelink V2X operating band n47, which is a TDD band, therefore, all the UEs in our simulation use half duplex as specified by the 3GPP standard [29].

4 Number of available RBs for a given bandwidth and 12 subcarriers per RB can be computed as: (*BW*(*Hz*)−*overhead*(*Hz*))/*SCS*(*Hz*) × 12. In this paper, we considered an overhead of 4% of the bandwidth, which is a typical value used in NR.

5 MCS 14 of NR MCS Table 1 and 10 RB long subchannel give a TBS of 348 bytes.

## ACRONYMS

$P_{\text{rsvp}}$: Resource Reservation Period
3GPP: 3rd Generation Partnership Project
5G: Fifth-Generation
5GAA: 5G Automotive Association
BSR: Buffer Status Report
BWP: Bandwidth Part
C-V2X: Cellular V2X
CDF: Cumulative Distribution Function
CP: Cyclic Prefix
CSI: Channel State Information
D2D: Device-to-Device
DMRS: Demodulation Reference Signals
DSRC: Dedicated Short Range Communications
eNB: Evolved Node B
EPS: Evolved Packet System
gNB: next-Generation Node B
HARQ: Hybrid Automatic Repeat Request
IE: Information Element
IP: Internet Protocol
ITS: Intelligent Transport System
KPI: Key Performance Indicator
LC: Logical Channel
LCID: Logical Channel Identifier
LTE: Long Term Evolution
MAC: Medium Access Control
MCS: Modulation Coding Scheme
MIMO: Multiple-Input Multiple-Output
mmWave: millimeter-wave
NAS: Non-Access Stratum
NDI: New Data Indicator
NR: New Radio
OFDM: Orthogonal Frequency Division Multiplexing
PDCP: Packet Data Convergence Protocol
PDU: Packet Data Unit
PHY: Physical
PIR: Packet Inter-reception Delay
PRR: Packet Reception Ratio
PSBCH: Physical Sidelink Broadcast Channel
PSCCH: Physical Sidelink Control Channel
PSFCH: Physical Sidelink Feedback Channel
PSSCH: Physical Sidelink Shared Channel
RB: Resource Block
RE: Resource Element
RLC: Radio Link Control
RRC: Radio Resource Control
RSRP: Reference Signal Received Power
RV: Redundancy Version
SCI: Sidelink Control Information
SCS: sub-carrier spacing
SI: Study Item
SLRRC: Sidelink Resource Reselection Counter
SPS: Semi-Persistent Scheduling
SUMO: Simulation of Urban MObility
TBS: Transport Block Size
TDD: Time Division Duplex
TDMA: Time-Division Multiple Access
TFT: Traffic Flow Template
TR: Technical Report
TS: Technical Specification
TTI: Transmission Time Interval
UE: User Equipment
UM: Unacknowledged Mode
V2I: Vehicle-to-Infrastructure
V2N: Vehicle-to-Network
V2P: Vehicle-to-Pedestrian
V2V: Vehicle-to-Vehicle
V2X: Vehicular-to-everything
WI: Work Item
